# Supplementary material for: Impact of involving people with dementia and their care partners in research: a qualitative study
Source: BMJ Open. 2020 Oct 27;10(10):e039321. doi: 10.1136/bmjopen-2020-039321 (PMC7592301; doi:10.1136/bmjopen-2020-039321)
Supplement: Supplementary data [file bmjopen-2020-039321supp001.pdf]

Supplementary file 1 – GRIPP2 short form

Section and topic	Item	Reported on page No
1: Aim	Report the aim of PPI in the study	2
2: Methods	Provide a clear description of the methods used for PPI in the study	3,4
3: Study results	Outcomes—Report the results of PPI in the study, including both positive and negative outcomes	6-9
4: Discussion and conclusions	Outcomes—Comment on the extent to which PPI influenced the study overall. Describe positive and negative effects	10,11
5: Reflections/critical perspective	Comment critically on the study, reflecting on the things that went well and those that did not, so others can learn from this experience	10,11
