# Supplementary material for: Impact of involving people with dementia and their care partners in research: a qualitative study
Source: BMJ Open. 2020 Oct 27;10(10):e039321. doi: 10.1136/bmjopen-2020-039321 (PMC7592301; doi:10.1136/bmjopen-2020-039321)
Supplement: Supplementary data [file bmjopen-2020-039321supp002.pdf]

Supplementary file 2 – PPI activity Monitoring Form

**RUG Site**	
Work Package	
Project lead/<br>Researcher	
Dates of Involvement	
Work identified	
PPI Input	
Feedback from RUG	
Action/s taken as a result of the feedback:	
Action not taken with a reason:	
Any other comments from Researcher / Project Manager	
