# Supplementary material for: Impact of involving people with dementia and their care partners in research: a qualitative study
Source: BMJ Open. 2020 Oct 27;10(10):e039321. doi: 10.1136/bmjopen-2020-039321 (PMC7592301; doi:10.1136/bmjopen-2020-039321)
Supplement: Supplementary data [file bmjopen-2020-039321supp004.pdf]

**Supplementary file 4 – Researchers interview topic guide**

1. What is your role within the SENSE-Cog Programme?

2. Which work package/s are you involved in?

3. What are your views on involving Research User Groups?

4. Have you been involved in any previous work involving Research User Groups?

5. What has been you experience of involving Research User Groups in the SENSE-Cog research programme:

    a) When were they involved?

    b) What were they involved in? and how did this help?

    c) Did you feel supported in involving Research User Groups?

    d) Did you feel confident in involving Research User Groups?

6. In what ways do you think the involvement of Research User Groups has impacted on your work in the SENSE-Cog programme?

7. Do you think you will be involving Research User Groups in other areas of your future work programmes?

8. Any other comments?



Miah J, *et al. BMJ Open* 2020; 10:e039321. doi: 10.1136/bmjopen-2020-039321
