# Supplementary material for: Impact of involving people with dementia and their care partners in research: a qualitative study
Source: BMJ Open. 2020 Oct 27;10(10):e039321. doi: 10.1136/bmjopen-2020-039321 (PMC7592301; doi:10.1136/bmjopen-2020-039321)
Supplement: Author's manuscript [file bmjopen-2020-039321.draft_revisions.pdf]

BMJ Open is committed to open peer review. As part of this commitment we make the peer review history of every article we publish publicly available.

When an article is published we post the peer reviewers' comments and the authors' responses online. We also post the versions of the paper that were used during peer review. These are the versions that the peer review comments apply to.

The versions of the paper that follow are the versions that were submitted during the peer review process. They are not the versions of record or the final published versions. They should not be cited or distributed as the published version of this manuscript.

BMJ Open is an open access journal and the full, final, typeset and author-corrected version of record of the manuscript is available on our site with no access controls, subscription charges or pay-per-view fees (http://bmjopen.bmj.com).

If you have any questions on BMJ Open's open peer review process please email info.bmjopen@bmj.com

BMJ Open

The impact of involving people with dementia and their care partners in research: A qualitative study

Journal:	*BMJ Open*
Manuscript ID	bmjopen-2020-039321
Article Type:	Original research
Date Submitted by the Author:	22-Apr-2020
Complete List of Authors:	Miah, Jahanara; University of Manchester, Division of Neuroscience and Experimental Psychology; Manchester University NHS Foundation Trust, Public Programmes Team<br>Parsons, Suzanne; Manchester University NHS Foundation Trust, Public Programmes Team<br>Lovell, Karina; The University of Manchester, The School of Nursing, Midwifery and Social Work<br>Starling, Bella; Manchester University NHS Foundation Trust, Public Programmes Team<br>Leroi, Iracema; Global Brain Health Institute, School of Medicine; University of Manchester, Division of Neuroscience and Experimental Psychology<br>Dawes, Piers; University of Manchester, Manchester Centre for Audiology and Deafness (ManCAD); Macquarie University, Department of Linguistics, Australian Hearing Hub
Keywords:	QUALITATIVE RESEARCH, Dementia < NEUROLOGY, PUBLIC HEALTH

SCHOLARONE™
Manuscripts

I, the Submitting Author has the right to grant and does grant on behalf of all authors of the Work (as defined in the below author licence), an exclusive licence and/or a non-exclusive licence for contributions from authors who are: i) UK Crown employees; ii) where BMJ has agreed a CC-BY licence shall apply, and/or iii) in accordance with the terms applicable for US Federal Government officers or employees acting as part of their official duties; on a worldwide, perpetual, irrevocable, royalty-free basis to BMJ Publishing Group Ltd ("BMJ") its licensees and where the relevant Journal is co-owned by BMJ to the co-owners of the Journal, to publish the Work in this journal and any other BMJ products and to exploit all rights, as set out in our licence.

The Submitting Author accepts and understands that any supply made under these terms is made by BMJ to the Submitting Author unless you are acting as an employee on behalf of your employer or a postgraduate student of an affiliated institution which is paying any applicable article publishing charge ("APC") for Open Access articles. Where the Submitting Author wishes to make the Work available on an Open Access basis (and intends to pay the relevant APC), the terms of reuse of such Open Access shall be governed by a Creative Commons licence – details of these licences and which Creative Commons licence will apply to this Work are set out in our licence referred to above.

Other than as permitted in any relevant BMJ Author's Self Archiving Policies, I confirm this Work has not been accepted for publication elsewhere, is not being considered for publication elsewhere and does not duplicate material already published. I confirm all authors consent to publication of this Work and authorise the granting of this licence.

For peer review only

Title page: The impact of involving people with dementia and their care partners in research: A qualitative study

Jahanara Miah[1,2] Jahanara.miah@manchester.ac.uk

Suzanne Parsons[2] Suzanne.Parsons@mft.nhs.uk

Karina Lovell[3] Karina.lovell@manchester.ac.uk

Bella Starling[2] Bella.Starling@mft.nhs.uk

Iracema Leroi[1,4] Iracema.leroi@tcd.ie

Piers Dawes[5,6] Piers.dawes@mq.edu.au *corresponding author

[1]University of Manchester, Division of Neuroscience and Experimental Psychology, Manchester, UK

[2]Manchester University NHS Foundation Trust, Public Programmes Team, Manchester, UK

[3]University of Manchester, The School of Nursing, Midwifery and Social Work, Manchester, UK

[4]Global Brain Health Institute, School of Medicine, Dublin, IE

[5]University of Manchester, Manchester Centre for Audiology and Deafness (ManCAD), Manchester, UK

[6]Macquarie University, Department of Linguistics, Australian Hearing Hub, Sydney, NSW, AUS

Keywords: Patient and Public Involvement, Dementia, Impact, Evaluation, European

Word count: 3908

For peer review only - http://bmjopen.bmj.com/site/about/guidelines.xhtml

The impact of involving people with dementia and their care partners in research: A qualitative study

Abstract

Objectives: We aimed to evaluate the impact of Patient and Public Involvement (PPI) at each stage of the research cycle in a dementia research program.

Design: We used monitoring forms and qualitative interviews with all participants to evaluate and record the impact of the research program's PPI at different stages of research.

Setting: We evaluated Research User Groups (RUGs) which were established to provide PPI support for the research program in multiple European sites (Manchester, Nice, Nicosia and Athens).

Participants: We purposively sampled RUG members (n=34) and researchers (n=13) who had participated in PPI activities. Inclusion criteria for the study were: (a) RUG members who had participated in the Research awareness training (RAT) and in PPI activities and had the capacity to consent; (b) researchers who involved RUGs in their work.

Results: *Impact on the research:* Changes to the study conduct were made as a result of the feedback from RUGs. These included prioritisation of clinical recommendations, the wording of study information and recruitment materials, the content and layout of the user interface for a computerised memory test, interpretation of intervention results and advice on dissemination avenues. *Impact on RUG members*: They reported that involvement had given them a sense of purpose and satisfaction. Their perception of health research changed from being an exclusive activity to one, which lay people, could have meaningful involvement. *Impact on researchers*: PPI was a new way of working and interacting with PPI members had given them insight into the impact of their work on people living with dementia.

Conclusions: PPI can have a substantial impact on dementia research and the people involved in the research. To justify the time and expense of PPI, the advantageous practical impacts of PPI should be systematically recorded and consistently reported.

Strengths and limitations of this study

- Patient and Public involvement (PPI) shows demonstrable impacts on research.

For peer review only - http://bmjopen.bmj.com/site/about/guidelines.xhtml

- The interaction between Research User Groups (RUGs) and researchers reveals gains in experiential knowledge and learning.
- Through its international reach, this study is an important contribution to the growing literature of PPI in dementia research.
- RUG interview data collected by the PPI coordinators may have biased into RUG participant responses.
- The RUG could have included people from more diverse backgrounds.

Introduction

Recognition that considering the views of end-users in health research improves the relevance and quality of health research has raised the profile of patient and public involvement (PPI).[1] PPI reflects the growing democratisation of health research and the importance of research impact. PPI ensures that research questions, conduct and outcomes are relevant and meaningful to patients and the public.[2]

In countries such as the UK, PPI is required by many funders,[3] as well as an ethical obligation.[4] Researchers are encouraged to involve patients and the public at all stages of the research cycle.[5] However, substantial challenges with the conception and implementation of PPI are still evident.[6-9] Although many health research studies acknowledge the beneficial impacts of PPI[7], various reviews highlight that the reporting of PPI is often of poor quality and evidence of impact is weak.[7-12] Thus, there is a clear need to demonstrate the impact of PPI on the research process more rigorously. Detailing the contribution made by PPI enables learning how to do PPI effectively and how to identify key indicators of impact.[13,14] In the context of the international multi-site dementia research programme, SENSE-Cog (www.sense-cog.eu), our aims were to: (i) identify the impact of PPI at each stage of the research cycle; (ii) provide a model for recording the impacts of PPI; and (iii) explore the experiences and perceptions of people with dementia, their care partners and dementia researchers of PPI in the research programme.

Context of the SENSE-Cog Patient and Public Involvement work

SENSE-Cog,[15] is a five-year (2016-2020) European research programme investigating the combined impact of ageing-related hearing and vision impairment on mental well-being. It involves the exploration of longitudinal datasets to understand the interplay of hearing, vision and cognitive

For peer review only - http://bmjopen.bmj.com/site/about/guidelines.xhtml

impairment,[16-18] the validation of assessments adapted for people with dementia and hearing and/or vision impairment,[19] and the evaluation of a home-based hearing and vision support intervention in improving quality of life for people with dementia.[20,21,22] As part of this programme, a dedicated work package on PPI[23] was embedded as a cross cutting theme (Figure 1) to involve older people with dementia and care partners. This involved establishing Research User Groups (RUGs) in Manchester (UK), Nice (France), Nicosia (Cyprus) and Athens (Greece) for PPI at each stage of the SENSE-Cog programme. Each RUG consisted of seven to nine RUG members, who were people with dementia and their care partners. RUG members were provided with research awareness training (RAT; outlined elsewhere [22]) to equip them with the skills and knowledge to guide their involvement in the research and to enable them to contribute to the research programme. Each RUG site was managed and supported by a local PPI coordinator in each of the four sites.[23] We reported all features of the PPI process according to the GRIPP2 short form recommendations[14] (Supplementary file 1) in this paper, and the full protocol involving the RUGs is published elsewhere.[23]

Figure 1: SENSE-Cog work package aims

The published protocol[23] (Supplementary file 2) outlining the PPI activities pertaining to the entire SENSE-Cog programme, was established by the RUGS at the outset of the research programme. Additional PPI activities were added at different stages on an 'as needed' basis by local RUGs and to include local contextualised content for the PPI activities. These activities were coordinated both centrally by the lead PPI coordinator based in Manchester, as well as locally by the site-specific coordinators. For the basic programme-wide PPI activities, the central PPI coordination team, RUG and cross-site researchers worked together to agree on the key objectives, formulate the PPI questions, and develop and revise the materials for the PPI activities. The central PPI coordinator supported the local RUGs and their PPI coordinators to implement and shape the activities in a manner that was appropriate and relevant to their local needs.

Methods

Patient and public involvement

A member of the Manchester RUG provided PPI on this paper to ensure it was informed by the perspectives of a person with experience of involvement in health research. PPI input was obtained

For peer review only - http://bmjopen.bmj.com/site/about/guidelines.xhtml

by a face-to-face consultation. The RUG member first reviewed the structure of the paper, including a rationale, the introduction, background and methods sections. The RUG member suggested the use of diagrams to illustrate the main findings and contributed ideas on how to categorise the findings. We acted on this by using a table to illustrate the key PPI activities in this paper. Second, the RUG member reviewed and commented on the draft paper. The RUG member supported the findings and approved the final paper. PPI in this paper provided the perspective of a person with experience of involvement in health research. The RUG member requested not to be named on this paper.

Monitoring and evaluating the impact of the SENSE-Cog PPI programme

We used two methods to monitor and evaluate the PPI work package within SENSE-Cog programme, (a) PPI monitoring forms (Supplementary file 3), throughout the research program; and (b) qualitative interviews conducted towards the end of the program.

- *Monitoring process:*

For all PPI activities, local PPI coordinators kept monitoring logs of the feedback from RUG members at each site (Figure 2). Types of feedback sent back to the research teams included: the wording of an adapted rating scale evaluating support care needs; the wording of participant information sheets; content and presentation of the intervention manual; aspects of the intervention itself (a multi-module intervention involving hearing and vision rehabilitation supported by a therapist); adaptation of a hearing aid guide for person with dementia (PwD) for the intervention manual; confirmed the researchers' analysis and refined the coding framework (Table 1). The completed PPI monitoring logs from the sites were sent to the central PPI coordinator in Manchester for recording purposes, and were then forwarded to the relevant work package research team for comments who recorded the actions taken to address the feedback on the monitoring form. All RUGs were given updates on the progress of the overall SENSE-Cog programme every 18 months to inform them of new developments and outputs and for the RUGs to witness how their feedback had shaped different aspects of the work. The PPI monitoring forms were examined by the central PPI coordinator and impacts and justifications for no action following RUG recommendations extracted and itemised in a table format (Table 1).

Figure 2: SENSE-Cog PPI model of working: monitoring and evaluation

- *Qualitative interviews:*

For peer review only - http://bmjopen.bmj.com/site/about/guidelines.xhtml

Participants for the qualitative sub-study

Participants for the impact evaluation were purposively sampled from two of the PPI working groups: (a) RUG members (n=34) who had participated in the RAT and in PPI activities and had the capacity to consent; (b) researchers who involved RUGs in their work. We aimed for a sample size of 6 RUG participants in each RUG site and 6 researchers across all sites to ensure representation and was determined by data saturation, the point when no new information was obtained. [24,25] Thirty-four RUG member were invited for interviews. Twenty-three RUG members responded and participated in the interviews. Eighteen researchers were approached for interviews, thirteen researchers responded and participated in the interviews. A total of thirty-six interviews were conducted (Table 2).

Table 2: Characteristics of participants

**RUG Site**	**Person with dementia**	**Care partner**	**Researcher**	**Researcher project manager**
Manchester	3	4	5	1
Nicosia	3	2	1	2
Nice	2	3	1	0
Athens	3	3	1	1
**Total**	**11**	**12**	**9**	**4**

Procedure for qualitative sub-study

We wrote two interview schedules, one for RUG participants (Supplementary file 4) and one for researchers (Supplementary file 5). The interview schedule for RUG participants was designed to understand their experience as a RUG member, participating in PPI activities and the perceived impact of their input on the research process. The interview schedule for researcher participants was developed to explore the researcher's view of PPI, their experience of working with RUGs and their perceived impact of PPI on the research.

Interviews with RUG participants, lasting about an hour each, were conducted between October 2018 and October 2019 by local PPI coordinators in each site. The interviews were conducted separately or in pairs as preferred by the person with dementia and care partners. Interviews with researcher

For peer review only

participants were conducted between January 2019 and September 2019 by the central PPI coordinator (JM) in English, either by phone of face-to-face.

Interviews with English speaking participants were audio-recorded and then transcribed verbatim. Interviews in Greek and French were transcribed verbatim in the local language and translated by the local PPI coordinators. Personal information was removed from transcripts.

Qualitative data analysis

Researcher and RUG data were pooled across sites and analysed. We undertook thematic analysis[26] to draw out the main characteristics of RUG members and researchers experiences. JM undertook the primary analysis, reading transcripts numerous times to develop open codes. Various levels of open coding took place from line-by-line coding. JM and SP independently examined the data to identify themes and reach consensus on interpretation. Themes were then assigned codes according to the Framework method[27] using NVivo software version 11 (QSR International, Doncaster, Australia). This method allows the data to be structured into a chart with columns and rows aiding the identification and grouping of text into relevant parts.

The coding framework for the RUG participants was emailed to local site PPI coordinators who were asked to provide input on a draft of the coding framework. Any additional themes identified by coordinators were added to the list of themes. Datasets were then re-analysed according to the final coding framework. We stopped data collection when no new information was being generated from analysis.[24]

Ethical approval

The study was conducted in Manchester, Nicosia, Nice and Athens. Participants provided informed written consent prior to the interview. The study received ethical approval from Manchester University Research Ethics Committee. Additional ethical approvals were sought and obtained for each study site (Nicosia, Nice, Athens), as relevant to local arrangements.

Results

Impact of PPI on research

For peer review only - http://bmjopen.bmj.com/site/about/guidelines.xhtml

PPI resulted in specific changes at all stages of the research from identifying and prioritizing the research agenda to dissemination (Table 1). In some cases, changes recommended by PPI were not implemented. Reasons that changes could not be implemented included scientific or regulatory requirements (e.g. not being able to make changes to validated psychometric tests, not being able to supply clinical diagnoses on the basis of tests given for a research study, not being able to shorten the participant information sheet due to ethical governance requirements) or limited financial or personnel resources (e.g. being able to produce a study newsletter only four times a year rather than more frequently).

RUG members' perspectives

Four themes were identified: facilitators to contributing, meaningful PPI, personal gains and perceived impact of involvement on research. In the following quotes, the site is reported first (i.e. 'Manchester', 'Nice', 'Nicosia' or 'Athens'). 'RUG (number)' refers to the individual RUG member being quoted. 'PwD' and 'care partner' refers to a person with dementia or a care partner for someone with dementia, respectively.

Facilitators to contributing in PPI

Participants discussed the benefits of group work. Group work was viewed as peer support for participants, which was seen as a valuable way to help understand each other's circumstances and needs to support each other. Participants reported that they had gained new knowledge and discussed the benefits of receiving the RAT for the role, highlighting that the structuring of the training alongside the involvement tasks as factors that helped them to contribute to research. RUG participants also reported that the support provided by the local PPI coordinators made them feel that they were making a valuable contribution. A participant commented on the importance of the *'human relationship' (Athens, RUG4, care partner)* with the coordinator.

For peer review only - http://bmjopen.bmj.com/site/about/guidelines.xhtml

Table 1: Summary of key PPI activities and outcomes linking to the stage of the research cycle

PPI activities for RUGs	Outcomes due to PPI input	PPI input not acted on
**Identifying and prioritising the research agenda**		
• International clinical practice recommendations: Recognition and management of hearing and vision impairment in dementia.<br><br>To contribute to development, relevance, and prioritisation the guidelines	-Support for clinical practice recommendations confirmed the relevance of the work.<br><br>-Prioritised recommendations used in the final document e.g. “Train GP’s to query if older patients have had their eyes/ ears tested”, “The benefits of wearing hearing aids should be highlighted to both PwD and care-partners, “reduce burden due to communication difficulties”<br><br>-Final recommendations endorsed by RUG members.	
**Design**		
• E-checker toolkit<br><br>To contribute to developing an online screening toolkit	-Instructions made clearer.<br>-Text size increased and incorporated throughout the interface for ease of access.<br>-instructions on how to proceed test added.<br>-The layout changed to make it more spontaneous, less formal and more inviting as suggested.<br>-Feedback after each test rather than all at the end.	-The toolkit cannot provide a clinical diagnosis as suggested by PPI because it is an unvalidated test used for research purposes only.

For peer review only

For peer review only - http://bmjopen.bmj.com/site/about/guidelines.xhtml

	-Result feedback format changed.<br>-Female voice included.<br>-Informant questionnaire changed from 'informant' to "a person who knows you well".<br>-Grey boxes changed to a brighter colour.<br>-Cheering for correct answers removed.	
• E-checker toolkit<br><br>User test E-checker. Provide feedback on appearance, usability, the feasibility of the E-checker.	-Font and layout changed to make it easier to see and use.<br>-Instructions simplified.	
• Paper participant Information Sheet (PIS)<br><br>• Audio-visual PIS<br><br>To provide comments on paper and audio-visual PIS	-Language simplified and spelling errors corrected on the paper version.<br>-Supplementary text changed alongside audio-voice on audio-visual PIS.	-Change to shorten the length of the paper participant information sheet could not be made due to ethics requirements.<br>-The voice-over on audio-visual participant information sheet was not changed to female voice due to limited resources.
• Sensory support Intervention<br><br>To comment on intervention design	-Inclusion criteria changed to include people with more advanced dementia.	

For peer review only - http://bmjopen.bmj.com/site/about/guidelines.xhtml

For peer review only

	-The number of visits reduced to minimise the burden on PwD and care partners.<br>-Recruitment team briefed to ensure they are clear to participants about the possible benefits of participating.<br>-Recruitment team briefed to ensure that people randomised to the Care as Usual group know how to access support for their impairment/s.	
• Public recruitment poster<br><br>To provide feedback on the presentation, readability, text, images, wording and layout of the poster	-'Dementia research' on title added to encourage/draw attention from target population.<br>-Clarified random allocation, specified as 'group 1 - Care as usual and group 2 – intervention.	
• Sensory Intervention diaries<br><br>To provide feedback on participant and study partners' diary	-Diaries were provided at each visit rather than together as a booklet at the end.	
• Communication guidance<br><br>To advise on contents of communication manual, and materials for use with participants and their study partners	-Communication manual supplemented with tips leaflet from Action on Hearing Loss.<br>-Training delivered to sensory support therapists included advice about adjusting the communication manual to fit with the current abilities/stage of the person with dementia, to maximise their co-operation and inclusion.	-The communication manual was not made into a storyboard format due to limited resources.

For peer review only - http://bmjopen.bmj.com/site/about/guidelines.xhtml

• Hearing aid leaflet<br><br>Contribute to developing a dementia-friendly hearing aid user guide	-Changed the leaflet size to A5.<br>-More white space created to make it easier to read.<br>-Font size increased.<br>-Instruction wording changed as suggested throughout the leaflet.<br>-The photo on a front-page removed and changed to hearing aid cartoon image.	
• Glasses care leaflet<br><br>Contribute to developing dementia-friendly glasses care guidance leaflets	-Changed the size, layout, font and wording of the leaflet.<br>-Cross and ticks used to indicate 'do's and don'ts'.<br>-The Sensory Support Therapists (SSTs) to clarify the cloth, wipes and use of glasses straps with the participants during their visits.	
• Interview questions<br><br>To advise on qualitative interview schedules on questions for PwD with hearing aids	- Language simplified to ask about people's experiences of using hearing aids.<br>-Confirmed the relevance of the questions.	
• Clinical questionnaires<br><br>To provide feedback on clinical questionnaires	-Spelling errors corrected.	
• RUD-Lite questionnaire		-Questions could not be directly changed on the RUD-lite questionnaire as it is a validated tool for the trial.

For peer review only - http://bmjopen.bmj.com/site/about/guidelines.xhtml

To advise on the meaning of RUD-lite questionnaire		
• Model validation<br><br>Contribute to developing the economic model validation	-Economic model shaped by RUG members experiences<br>-RUG members confirmed research reports concerning health economic impacts of dementia.	
**Analysing & interpreting**		
• Main trial qualitative data interpretation<br><br>Assist with data analysis on selected transcripts to identify codes	- The codes identified by the RUGs confirmed the researchers' analysis and helped to refine the 'coding tree', as well as identify additional themes that were not identified by the researchers.	
• Modelling the impact of sensory interventions on cognitive health	-Discussion of RUG experiences confirmed the research team's interpretation of the results.	
**Dissemination**		
• Dissemination strategy<br><br>Advise on the dissemination plan	-Public/social events incorporated into the dissemination plan.<br>-Local and national stakeholder groups included in the dissemination list for the newsletter and network list.	

For peer review only - http://bmjopen.bmj.com/site/about/guidelines.xhtml

	-YouTube videos produced online about current research programme and findings.	
• The impact of sensory interventions on cognitive health<br><br>Advise on dissemination routes for the findings	-Dissemination of findings to a wider public audience via national newspapers, including Daily Mail, The Times.	
• Nicosia public engagement event<br><br>Provide feedback on content and structure of engagement event	-Changes were made to the presentation agenda to include care partners' perspectives, information, and support for care partners.<br>-2 RUG members gave presentations about their involvement in research.	
• Nice public engagement event<br><br>Provide feedback on content and structure of engagement event	-Wording, layout and language simplified on the presentation slides.<br>- Workshop included an audience discussion on the concept of PPI.	
• Newsletter<br><br>Advise on name of the newsletter, content and format of the project newsletter	-Newsletter named 'SENSE-Cog news'.<br>-Suggestions concerning the content were incorporated to include updates on the studies, ongoing activities within SENSE-Cog with a section on each study site, biographic details of research teams and forthcoming events.<br>-A section on RUGs perspectives included.	-Newsletter published quarterly and not every 2 months as suggested due to limited resources.

For peer review only - http://bmjopen.bmj.com/site/about/guidelines.xhtml

• INVOLVE conference 2017<br><br>Provide feedback on a planned conference presentation	-Changes were made to the order of the slides to make it easier to follow.<br>-Additional handout materials included for the presentation to provide more information about the research.	
• PPI protocol paper<br><br>Contribute to the plain English summary section	-Changes were made to the text to simplify the wording and the approved the plain English summary.	
• PPI scoping review<br><br>Provide feedback on the main findings of the review	-Discussions and recommendations on findings incorporated in the findings section of the paper.	
• AAIC conference poster presentation<br><br>Advise on the contents and presentation of conference paper	-Layout changed on the poster to include more white space to make it visibly appealing, wording simplified, and content re-arranged to make the materials easy to read.<br>-RUGS chose photos of PPI activity to be included on the poster.	
• Age and accessibility workshop<br><br>Provide feedback on a planned workshop presentation	- More details about the support provisions for RUGs were included in the presentation slides.<br>- Wording simplified and texts size increased.<br>-Video of a RUG member talking about her experience of involvement included in the presentation.	

For peer review only - http://bmjopen.bmj.com/site/about/guidelines.xhtml

• NIHR PPI and inclusion workshop<br><br>Provide feedback on a planned workshop presentation	- Wording simplified.<br>-Video of a RUG member talking about her experience of involvement included in the presentation.	
• SENSE-Cog website publicity materials<br><br>Advise on content and images for websites and publicity materials	- Chose photographs for use in the study website and other public-facing materials.<br>-Wording on the website changed to simplify language. The term ‘elderly’ changed to ‘older European citizens’ and ‘seniors’.	

For peer review only - http://bmjopen.bmj.com/site/about/guidelines.xhtml

Meaningful PPI

Interaction with researchers and having researchers listen to RUG member's opinions to was reported as an important aspect. Receiving feedback was also highlighted as an important factor in letting RUG members know that their contribution was having a tangible impact:

"I feel that our communication was quite satisfactory and apart from that we had feedback. We said something and they 'listened' to it and we move forward, they tell you a little more, something like that….Yes, for sure. Otherwise, I wouldn't be here today. It's a good feeling, to be heard. You gain this feeling that you are not alone and that there are people that care for you, and that what you say can go further" (Athens, RUG5, care partner*)*

Personal benefits of PPI

Face-to-face group meetings provided RUG members the opportunity to share issues with others in similar position. RUG members reported that the experience personally benefited them in terms of gaining new knowledge, interacting with others, giving advice and feeling that they were making a worthwhile contribution. RUG participants felt their involvement exceeded their expectations, due to continuous involvement and training in research awareness:

"Throughout the meetings we felt that what we learned is very important for the lives of individuals. I felt the role was more than was described (role of RUG member), because we participated (learned) in many task trying to find solutions that we have to resolve." (Athens RUG6, PwD)

Perceived impact of involvement on research

RUG members talked about their involvement in dissemination activities, particularly in countries other than the UK. RUG members talked about how providing their perspective provided researchers with a better understanding of the impact of dementia that informed research decisions. RUG members emphasised their contribution in making written materials more accessible for participants and suggesting changes in the interventions and tools developed by the research team. RUG members reported that incremental changes informed by PPI could make a difference to practical outcomes:

For peer review only - http://bmjopen.bmj.com/site/about/guidelines.xhtml

"Well it's just one factor in the complicated chain of conditions that lead to the outcome. So it's not going to have any direct impact on any individual people, but every time a member of the group makes a contribution, it's adding a little bit more information to the whole package of information that's going to improve the lives of the end users in the long term." (Manchester, RUG1, ex-care partner)

Researchers' perspectives

Four themes were identified: limited experience of PPI, acceptance and importance of PPI, beneficial impacts of involvement and challenges. In the following quotes, the site is reported first (i.e. 'Manchester', 'Nice', 'Nicosia' or 'Athens') then the researcher number. 'PM' refers to project managers.

Limited experiences of PPI

Of the 13 researchers interviewed, 10 had no previous experience of systematically obtaining patient and public input to planning or conduct of research:

"So it was the first time we've heard about it, on this project. So it took us all a while to understand it, but I mean by going through it and by doing it we understood the importance of it." (Nicosia, R3)

Acceptance and importance of PPI

There was general acceptance of PPI by researchers and acknowledgement of the importance of the PPI integrated into the research programme. Researchers reported that PPI provided them with the perspective of living with dementia:

"I think it's very useful and I think it gives a perspective to research that we needed to have, because we kind of tend to, you know, sort of take the feeling out of our day to day research and when you have the actual patients explain to them rather than peers, it does give us a different perspective" (Manchester, PM9)

Beneficial impacts of involvement

For peer review only - http://bmjopen.bmj.com/site/about/guidelines.xhtml

Researchers talked about the beneficial impacts of PPI, including refining content and wording of public-facing information and identifying issues not considered by the researchers. One researcher commented that PPI provided insight into what patients and the public think of their work. Researchers valued PPI with respect to understanding the impacts of dementia on daily life and how these impacts should be captured in health economic measurement, ensuring the appropriateness and relevance of health economic measures:

"We were trying to confirm that using dependence as some measure of progression in our economic model would be appropriate and having spoken to the RUG members, they, kind of, confirmed that. And then there were some, kind of, hallmark things that stood out that they all, kind of, agreed on in their discussions.........It has helped in terms of model of validation." (Man, R12)

Some researchers also talked about the emotional impact of listening to RUG members' experiences, which were distressing at times, particularly for researchers not used to patient contact. Researchers reported that their interaction with RUG members led to researchers changing future practice, such as taking patient perspectives into account in planning and prioritising research:

"I've written numerous grant proposals since SENSE-Cog began, and in some of them I've included…wherever it makes sense, I've included a research utilisation for a patient and public involvement group." (Nicosia, PM8)

Challenges

Researchers talked about challenges, such as PPI suggestions not being able to be implemented because of scientific reasons or resource limitations. Another researcher highlighted the importance of identifying points of research design where PPI would have a critical impact and reported a missed opportunity concerning selection of outcome measures for an intervention study due to time constraints in development of the study protocol.

Discussion:

For peer review only

For peer review only - http://bmjopen.bmj.com/site/about/guidelines.xhtml

PPI is proposed as an important means of enhancing the relevance and impact of research.[5,28] However, while the descriptive literature on PPI is growing, there is still little evidence for how PPI actually impacts on research and there has been no clear methodology for recording impact.[6-8,11] Here, we reported a method for capturing the impact of PPI in a multi-centre European research project, outlining the impact of PPI at each stage of the research process. We also provided qualitative perspectives of RUG members and researchers concerning their views on the impact of involving people with dementia and care partners in research.

A number of key changes were made to different aspects of the research program at various points following PPI advice. PPI contributed substantive input to conception, design, recruitment, interpretation of results and dissemination of the research. However, some PPI feedback could not be acted on for the reasons outside the research team's control due resource restrictions, lack of time or compromising validated psychometric measures, similar to restrictions on implementing PPI advice reported elsewhere[10,29]. Without ongoing advice from the RUGs to the research teams, researchers may not have made appropriate changes on many aspects of the research design to take account of the experience of living with dementia. In addition to concrete changes in the research following RUG input, RUG members reported various personal benefits in contributing to research, including feeling that they were making a useful contribution to research. Researchers reported that the RUG members input gave them an important personal perspective on the relevance and impact of their research for people living with dementia.

Limitations

RUG interview data were collected by the coordinators who were themselves SENSE-Cog project researchers, rather than by an independent researcher. Data collection by SENSE-Cog project researchers may have resulted in responses being overly positive.[26,27]

Although research involvement opportunities for RUGs were advertised via contact with local charities, social organisations and support groups, we received few queries about involvement and no queries from anyone from minority ethnic communities. The RUG groups may not have reflected the diversity within the general community, and this may have limited the perspectives that RUGs were able to offer and of the impact of PPI in this project. Using local community leaders or organisations to access more diverse communities could have increased recruitment from minority groups.[30-32] Future research could explore views of older adults from minority ethnic background and diverse communities in PPI.

For peer review only - http://bmjopen.bmj.com/site/about/guidelines.xhtml

For our qualitative work, we only sought the opinions of researchers who were involved in a project that contained a strong element of PPI. The researchers in this project may have been more favourably disposed to PPI than European researchers in general. Another limitation is that RUGs were not involved in the initial conception or planning of the study. Ideally, PPI should occur from the point of conception of a research program.

PPI in research has potential benefits, but it does require substantial resources that could otherwise be spent elsewhere. Although this paper highlighted the impact of PPI on the research and documented positive experiences of the RUG members and researchers, we cannot be sure of the impact of these changes on research outcomes (for example, if fewer participants would have been recruited if recruitment materials had not been altered following PPI input). An understanding of the benefit and cost utility of PPI would require a controlled evaluation (for example, recruitment with versus without PPI input on recruitment materials). There is some evidence from controlled evaluations that PPI does have tangible impact: a systematic review on the impact of PPI on enrolment and retention in clinical trials reported that PPI considerably improved the likelihoods of a patient joining a trial compared to recruitment strategies that did not include PPI.[33] However, because recruitment strategies that had used PPI also included non-PPI components, it was difficult to establish the contribution of PPI versus other factors.

Conclusion

This study provides a methodology for recording the impact of PPI in multi-centre health research using systematic ongoing monitoring and feedback throughout the research program, followed by detailed qualitative exploration of PPI contributors' and researchers' views. To justify the time and expense of PPI, the impacts and benefits of PPI should be systematically recorded and reported for all health research that makes use of PPI.

Further information about SENSE-Cog is available at www.sense-cog.eu

For peer review only - http://bmjopen.bmj.com/site/about/guidelines.xhtml

References

1. Tinetti ME, Basch E. Patients' responsibility to participate in decision making and research. *JAMA.* 2013;309:2331–2332.
2. Boote J, Wong R, Booth A. Talking the talk or walking the walk? A bibliometric review of the literature on public involvement in health research published between 1995 and 2009. *Health Expect.* 2015;18:44–57.
3. Department of Health. *Research governance framework for health and social care* London2005.
4. Iliffe S, McGrath T, Mitchell D. The impact of patient and public involvement in the work of the Dementias & Neurodegenerative Diseases Research Network (DeNDRoN): Case studies. *Health Expectations.* 2013;16(4):351–361.
5. INVOLVE. *Briefing notes for researchers: involving the public in NHS, public health and social care research.* 2012.
6. Staniszewska S, Brett J, Mockford C, Barber R. The GRIPP checklist: strengthening the quality and transparency of reporting for patient and public involvement in research. *Int J Technol Assess Health Care.* 2011;27:391–399.
7. Staniszewska S, Denegri S, Matthews R, V. M. Reviewing progress in public involvement in NIHR research: developing and implementing a new vision for the future. *BMJ Open.* 2018;8.
8. Miah J, Dawes P, Leroi I, Starling B, Edwards S, Parsons S. Patient and public involvement in dementia research in the European Union: a scoping review. *BMC Geriatrics.* 2019;19.
9. Bethell J, Commisso E, Rostad HM, et al. Patient engagement in research related to dementia: A scoping review. *Dementia.* 2018;17(8):944-975.
10. Brett J, Staniszewska S, Mockford C, al. e. Mapping the impact of patient and public involvement on health and social care research: a systematic review. *Health Expectations.* 2014;17(5):637–650.
11. Wilson P, Mathie E, Keenan J, et al. ReseArch with patient and public invOlvement: a RealisT evaluation - the RAPPORT study. *Health Serv Deliv Res.* 2015.
12. Mockford C, Staniszewska S, Griffiths F, Herron-Marx S. The impact of patient and public involvement on UK NHS health care: a systematic review. *Int J Qual Health Care.* 2012;24(1):28–38.
13. Evans D, Coad J, Cottrell K, et al. Public involvement in research: assessing impact through a realist evaluation. *Health Serv Del Res.* 2014.
14. Staniszewska S, Brett J, Simera I, et al. GRIPP2 reporting checklists: tools to improve reporting of patient and public involvement in research. *BMJ.* 2017;358.
15. SENSE-Cog project https://www.sense-cog.eu/. Accessed August 17, 2019.
16. Maharani A, Dawes P, Nazroo J, Tampubolon G, Pendleton N, Sense-Cog WP1 group. Visual and hearing impairments are associated with cognitive decline in older people. *Age and ageing.* 2018;47(4):575-581.
17. Maharani A, Dawes P, Nazroo J, Tampubolon G, Pendleton N, SENSE-Cog WP1 group. Cataract surgery and age-related cognitive decline: A 13-year follow-up of the English Longitudinal Study of Ageing. *PloS one.* 2018;13(10).
18. Maharani A, Pendleton N, Leroi I. Hearing Impairment, Loneliness, Social Isolation, and Cognitive Function: Longitudinal Analysis Using English Longitudinal Study on Ageing. *The American journal of geriatric psychiatry.* 2019 27(12):1348-1356.
19. Dawes P, Wolski L, Himmelsbach I, Regan J, Leroi I. Interventions for hearing and vision impairment to improve outcomes for people with dementia: a scoping review. *International psychogeriatrics.* 2019 31:203-221.

For peer review only

For peer review only - http://bmjopen.bmj.com/site/about/guidelines.xhtml

20. Hooper E, Simkin Z, Abrams H, et al. Feasibility of an Intervention to Support Hearing and Vision in Dementia: The SENSE-Cog Field Trial. *Journal of the American Geriatrics Society.* 2019 67(7):1472-1477.
21. Regan J, Frison E, Collin F, et al. Individualised sensory intervention to improve quality of life in people with dementia and their companions (SENSE-Cog trial): study protocol for a randomised controlled trial. *Trials.* 2019;20(80).
22. Miah J, Dawes P, Leroi I, Starling B, Lovell K, Parsons S. Evaluation of a research awareness training programme to support research involvement of older people with dementia and their care partners. In. *Health Expectations, Manuscript in preparation*2019.
23. Miah J, Dawes P, Leroi I, Parsons S, Starling B. A protocol to evaluate the impact of involvement of older people with dementia and age-related hearing and/or vision impairment in a multi-site European research study. *Research Involvement and Engagement.* 2018;4.
24. Guest G, Bunce A, Johnson L. How many interviews are enough? An experiment with data saturation and variability. *Field Methods.* 2006;18:59-82.
25. O'Reilly M, Parker N. Unsatisfactory saturation: A critical exploration of the notion of saturated sample sizes in qualitative research. *Qualitative Research Journal.* 2012;1(18).
26. Creswell J, Hanson W, Plano V, Morales A. Qualitative Research Designs Selection and Implementation. . *The Counseling Psychologist.* 2007;35:236-264.
27. Ritchie J, Lewis J. *Qualitative research practice: a guide for social science students and researchers.* . London: Sage; 2003.
28. Ocloo J, Matthews R. From tokenism to empowerement: progressing patient and public involvement in healthcare improvement. *BMJ Qual Saf.* 2016;25:626–632.
29. Domecq JP, Prutsky G, Elraiyah T, et al. Patient engagement in research: a systematic review. *BMC Health Serv Res.* 2014.
30. Rojas-García A, Turner S, Pizzo E, Hudson E, Thomas J, R. R. Impact and experiences of delayed discharge: a mixed-studies systematic review. *Health Expect.* 2017;2(1):41–56.
31. Ismail MM, Gerrish K, Naisby A, Salway S, P. C. Engaging minorities in researching sensitive health topics by using a participatory approach. *Nurse Res.* 2014;22(2):44-48.
32. Dudley L, Gamble C, Preston J, et al. What difference does patient and public involvement make and what are its pathways to impact? Qualitative study of patients and researchers from a cohort of randomised clinical trials. *PLoS One.* 2015;10(6).
33. Crocker JC, Ricci-Cabello I, Parker A, et al. Impact of patient and public involvement on enrolment and retention in clinical trials: systematic review and meta-analysis. *BMJ.* 2018;28.

Acknowledgments

The authors would like to acknowledge the people working with us as part of Research User Groups and SENSE-Cog colleagues coordinating the Research User Groups in the UK, France, Cyprus and Greece.

Funding

This protocol paper is part of Work Package 5 of the SENSE-Cog project, which has received funding from the European Union's Horizon 2020 research and innovation programme under grant agreement

For peer review only - http://bmjopen.bmj.com/site/about/guidelines.xhtml

No. 668648. Piers Dawes, Suzanne Parsons and Bella Starling are supported by the National Institute for Health Research Manchester Biomedical Research Centre.

Author Contribution

IL, PD, KL, BS contributed to the conception of the study. JM, PD and SP contributed to the design of the study. JM wrote the first draft and was involved in the editing of the manuscript, which was critically reviewed by IL, KL, PD and SP and approved the final manuscript.

Disclaimer

The funding body has no part in study design, data collection, data analysis, data interpretation, or manuscript writing.

Competing interests

The authors declare that they have no competing interests.

Ethics approval

Ethical Approval was approved by the University of Manchester's Research Ethics Committee UREC: 2017-0627-2142

Data sharing statement

Interviews data from this study are personally identifiable for RUG members and researchers, and are therefore not available for data sharing

Patient consent for publication

Obtained

For peer review only - http://bmjopen.bmj.com/site/about/guidelines.xhtml

For peer review only

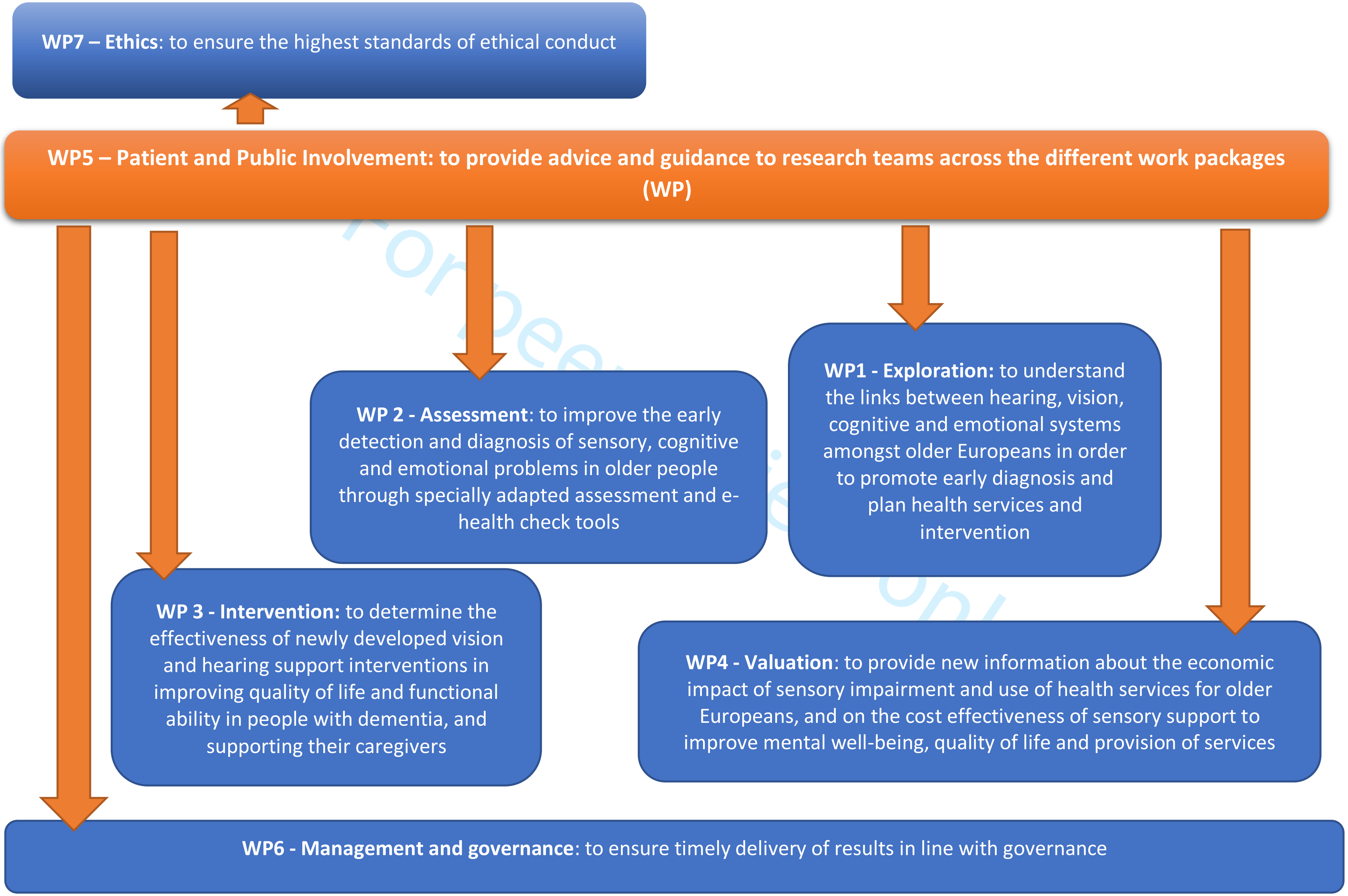
WP7 – Ethics: to ensure the highest standards of ethical conduct
WP5 – Patient and Public Involvement: to provide advice and guidance to research teams across the different work packages (WP)
WP 2 - Assessment: to improve the early detection and diagnosis of sensory, cognitive and emotional problems in older people through specially adapted assessment and e-health check tools
WP1 - Exploration: to understand the links between hearing, vision, cognitive and emotional systems amongst older Europeans in order to promote early diagnosis and plan health services and intervention
WP 3 - Intervention: to determine the effectiveness of newly developed vision and hearing support interventions in improving quality of life and functional ability in people with dementia, and supporting their caregivers
WP4 - Valuation: to provide new information about the economic impact of sensory impairment and use of health services for older Europeans, and on the cost effectiveness of sensory support to improve mental well-being, quality of life and provision of services
WP6 - Management and governance: to ensure timely delivery of results in line with governance

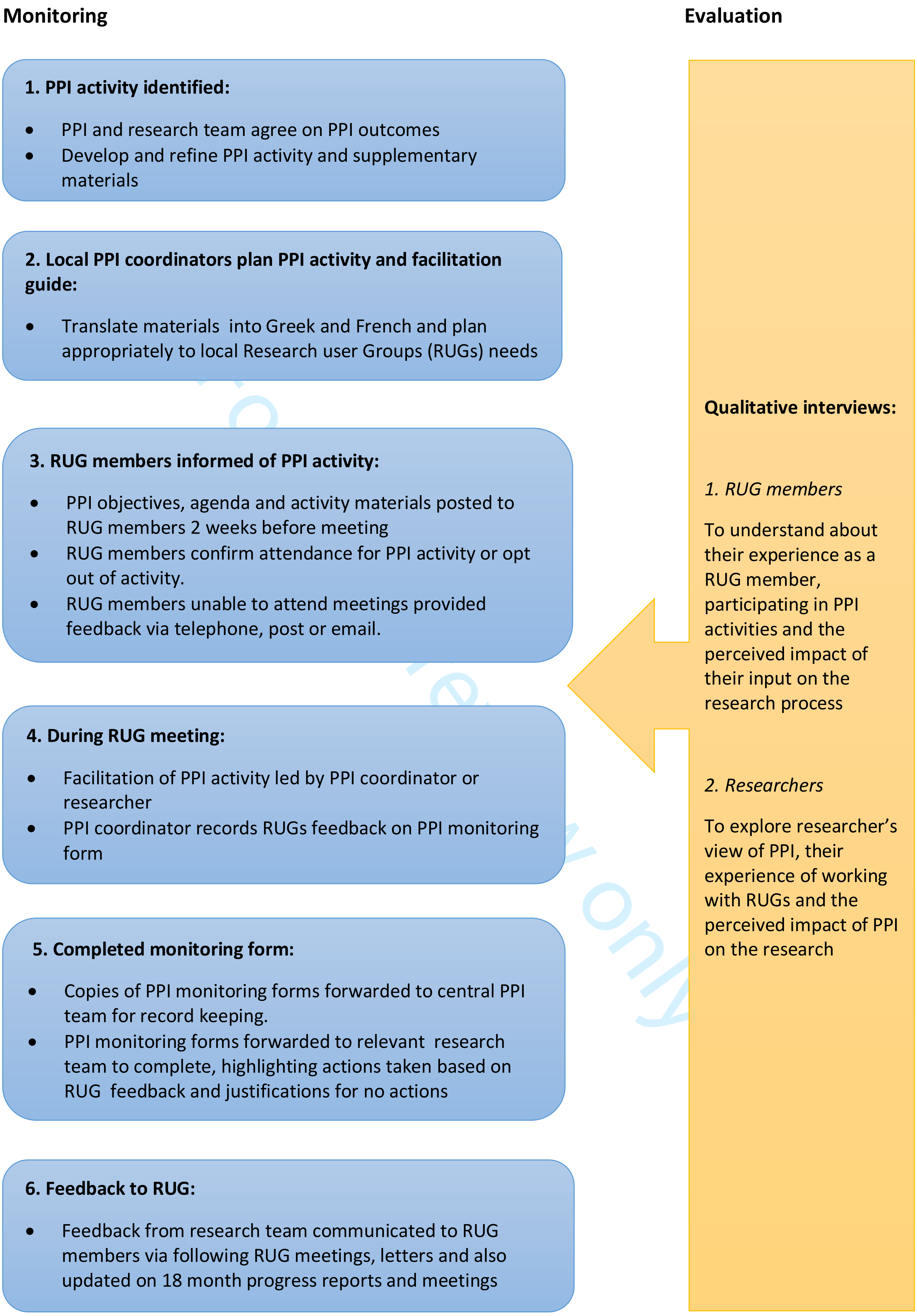
Monitoring
1. PPI activity identified:
PPI and research team agree on PPI outcomes
Develop and refine PPI activity and supplementary materials
2. Local PPI coordinators plan PPI activity and facilitation guide:
Translate materials into Greek and French and plan appropriately to local Research user Groups (RUGs) needs
3. RUG members informed of PPI activity:
PPI objectives, agenda and activity materials posted to RUG members 2 weeks before meeting
RUG members confirm attendance for PPI activity or opt out of activity.
RUG members unable to attend meetings provided feedback via telephone, post or email.
4. During RUG meeting:
Facilitation of PPI activity led by PPI coordinator or researcher
PPI coordinator records RUGs feedback on PPI monitoring form
5. Completed monitoring form:
Copies of PPI monitoring forms forwarded to central PPI team for record keeping.
PPI monitoring forms forwarded to relevant research team to complete, highlighting actions taken based on RUG feedback and justifications for no actions
6. Feedback to RUG:
Feedback from research team communicated to RUG members via following RUG meetings, letters and also updated on 18 month progress reports and meetings
Evaluation
Qualitative interviews:
1. RUG members
To understand about their experience as a RUG member, participating in PPI activities and the perceived impact of their input on the research process
2. Researchers
To explore researcher’s view of PPI, their experience of working with RUGs and the perceived impact of PPI on the research

Supplementary file 1 – GRIPP2 short form

Section and topic	Item	Reported on page No
1: Aim	Report the aim of PPI in the study	2
2: Methods	Provide a clear description of the methods used for PPI in the study	3,4
3: Study results	Outcomes—Report the results of PPI in the study, including both positive and negative outcomes	6-9
4: Discussion and conclusions	Outcomes—Comment on the extent to which PPI influenced the study overall. Describe positive and negative effects	10,11
5: Reflections/critical perspective	Comment critically on the study, reflecting on the things that went well and those that did not, so others can learn from this experience	10,11

A protocol to evaluate the impact of involvement of older people with dementia and age-related hearing and/or vision impairment in a multi-site European research study

Jahanara Miah[1,3] Jahanara.miah@manchester.ac.uk *corresponding author

Piers Dawes[2] Piers.dawes@manchester.ac.uk

Iracema Leroi[1] Iracema.leroi@manchester.ac.uk

Suzanne Parsons[3] Suzanne.parsons@mft.nhs.uk

Bella Starling[3] Bella.starling@mft.nhs.uk

[1]Division of Neuroscience and Experimental Psychology, University of Manchester, Jean McFarlane Building, Oxford Road, Manchester M13 9PL, UK

[2]Manchester Centre for Audiology and Deafness (ManCAD), Manchester Academic Health Science Centre, University of Manchester, Oxford Road, Manchester M13 9PL, UK

[3]Public Programmes Team, Research and Innovation Division, Manchester University NHS Foundation Trust and The University of Manchester, 29 Grafton Street, Manchester, M13 9WU

For peer review only - http://bmjopen.bmj.com/site/about/guidelines.xhtml

Plain English Summary

Involving older people with dementia in research is increasingly recognised as important to ensure that research is relevant and beneficial for older people with dementia. But researchers need to know how best to involve older people with dementia and to be able to show the benefits of involving older people with dementia in dementia research.

This paper describes a research plan to explore the involvement of older people with dementia and age-related hearing and/or vision impairment in a European research project investigating the combined impact of dementia with hearing and/or vision impairment. We set up four Research User Groups (RUGs) of older people with dementia with age-related hearing and/or vision impairment and their carers based in the UK, France, Cyprus and Greece to advise our researchers. We provided training to group members to support their input to the research.

We will use a questionnaire and interview people in our RUGs to understand what they thought of the training and their experiences of being part of the RUG. We will also interview researchers to understand if they thought the advice from the groups was useful.

This study will help us to understand how to effectively involve older people with dementia and age-related hearing and/or vision impairment in research and what the benefits of involving older people with dementia in research are.

For peer review only

For peer review only - http://bmjopen.bmj.com/site/about/guidelines.xhtml

Abstract

Background

Research to prevent and treat dementia is an international priority. Involvement of older people with dementia in the research is important to ensure the relevance and utility of the research outcomes in clinical practice to them. Efforts to involve such people in research are growing due to increased recognition of the usefulness of incorporating the views of older people with dementia into the research process. Research User Groups (RUGs) of older people with dementia and carers for people with dementia were set up in UK (Manchester), France (Nice), Cyprus (Nicosia) and Greece (Athens) to advise on the research. We report a protocol for a study which aims to evaluate i) the perceptions of RUG members of the usefulness of Research Awareness Training that was provided to support their involvement in the research and ii) perceived impacts of the involvement of older people with dementia and age-related hearing and/or vision impairment on research from the point of view of RUG members and researchers.

Methods

Both qualitative and quantitative methods will be used to evaluate the acceptability, appropriateness and satisfaction with Research Awareness Training and the perceived impact of involvement of RUGs on research. Focus groups interviews with RUG members and one to one interviews with both RUG members (n=24) and researchers (n=6) will be conducted to understand the perceived impacts of patient and public involvement on research from the point of view of older people with dementia, carers and researchers. Any comparative differences in cultural, attitudinal and environmental differences between RUGs in outcomes of training and impact across the four European sites will be reported.

Discussion

For peer review only - http://bmjopen.bmj.com/site/about/guidelines.xhtml

This study is unique in its exploration of the impact of the involvement of older people with dementia and age-related hearing and/or vision impairment in a large multi-site European dementia research study. This work will be crucial in informing understanding of how to effectively involve older people with dementia and age-related hearing and/or vision impairment and carers in dementia research to ensure research addresses the needs and priorities of older people with dementia and age-related hearing and/or vision impairment.

Keywords: Patient and Public Involvement, Older people, Dementia, Hearing, Vision, Europe, Research User Groups, Protocol

Background

Research to prevent and treat dementia as well as improve quality of life for people with dementia is an international priority [1]. Involvement of people with dementia in research is important to ensure the relevance and utility of the research outcomes in clinical practice [2, 3]. There is increasing recognition of the need to involve people with dementia in research due to an increased focus on person-centred care and the attendant requirement to take the views of people with dementia into account [4,5,6]. There is also evidence to suggest the involvement of people with dementia in research has an impact on research, researchers, the organisation and the person involved in the research [7]. Consequently, there is growing interest in understanding how to involve people with dementia as active partners in research [4,5,6].

The rationale for patient and public involvement in research in general is that people with lived experience of a health condition or treatment can offer valuable perspectives to the research process [8,9]. INVOLVE, the coordinating centre for patient and public involvement in the UK's National Health Service defines involvement as "research being carried out 'with' or 'by' members of the public rather than 'to', 'about', or 'for them'" [8]. Involvement can take a number of forms

For peer review only - http://bmjopen.bmj.com/site/about/guidelines.xhtml

including consultation (e.g. being involved in prioritising research), collaboration (e.g. acting as an advisor on a research project) or user control (e.g. acting as a service user researcher and carrying out the research) [8]. People with dementia may be involved in all stages of the research process [6,8,9]. For example, people with dementia may be involved in identifying research priorities relevant to patients and carers [10,11], developing funding applications [12], enhancing the relevance and feasibility of research to develop effective interventions [13], identifying potential barriers at an early stage in the research [14,15], contributing to the interpretation of findings [16,17], and the dissemination of research results [18,19]. Being involved in research may also provide benefits to people with dementia and their carers including increasing knowledge, enhancing skills, developing networks and new opportunities for involvement [20], meeting with and hearing the opinions of others with dementia [19,21], improving self-confidence, and offering opportunities to contribute to dementia research in a forum where the views of people with dementia are valued [21].

Study context

This study is being conducted as part of a larger project, 'SENSE-Cog', which aims to investigate the combined impact of dementia and age-related hearing and/or vision impairment and to develop new tools to improve quality of life for older Europeans [22]. SENSE-Cog is funded by the European Research Council's Horizon 2020 research and innovation programme. The project started in January 2016 and will run until December 2020. Involvement of older people with dementia in SENSE-Cog is a significant part of the overall work programme (Figure 1).

Figure 1

For peer review only - http://bmjopen.bmj.com/site/about/guidelines.xhtml

This paper focuses on SENSE-Cog work package five, 'patient and public involvement'. Older people with dementia and age-related hearing and/or vision impairment and carers are involved in SENSE-Cog research via Research User Groups (RUGs). RUG membership was constrained to people over the age of 65 years to reflect the target population of the SENSE-Cog research study. RUGs are groups of people who are brought together and supported to give their views and input into SENSE-Cog research. Four RUGs have been established at SENSE-Cog partner sites in Manchester, Nice, Nicosia and Athens. RUGs consist of 7-10 older people with dementia and age-related hearing and/or vision impairment and/or their carers.

Prior to recruiting RUG members, we consulted with a group of older people with dementia and their carers affiliated with Manchester Institute for Collaborative Research on Ageing (MICRA) in a focus group discussion to comment on a draft recruitment flyer for RUG members, we asked participants to comment on the wording, presentation and readability of the flyer. We also asked them to advise us on where to advertise the flyers, and on considerations when setting up meetings for older people with dementia and age-related hearing and/or vision impairment. The draft flyer was revised following the focus group recommendations and translated into French and Greek for the RUG sites in Nice, Nicosia and Athens. The flyers were advertised locally in each RUG site through local organisations and networks working with older people with dementia and older people with dementia and age-related hearing and/or vision impairment. For example, for the Manchester RUG recruitment, flyers were distributed via an email list members of the general public interested in aging research, local memory clinics, and charity organisations including the Alzheimer's Society, Age UK, Manchester Dementia Action Alliance and Action on Hearing Loss. We received relatively few queries about involvement in general and no queries from anyone from Black, Asian and minority ethnic or underserved communities. On reflection, this recruitment method was not very effective in reaching a diverse range of potential participants. A more targeted community outreach approach such as in-person talks to local dementia support groups,

For peer review only

For peer review only - http://bmjopen.bmj.com/site/about/guidelines.xhtml

organisations working with Black, Asian and minority ethnic or underserved communities could have been a better method of recruiting a more diverse RUG.

For all RUGs, introductory meetings were held to allow members to decide on how the group should operate and function, e.g. the frequency, length and format of meetings and training delivery. All RUGs are supported and managed by a local patient and public involvement coordinator. Local patient and public involvement coordinators were identified among the research team in each study site. Patient and public involvement coordinators have a background in research with people with dementia, and have experience of working with older adults in research settings. All patient and public involvement coordinators speak English, and communicate in English with the patient and public involvement work package coordinator in Manchester, UK. The role of the patient and public involvement coordinator is to arrange meetings, liaise with researchers and develop activities to support the groups' involvement in the research.

RUGs meet face-to-face every 3 months, as well as ad-hoc individual meetings with up to 2 RUG members (with the option of postal, telephone, e-mail communication, or in person) for any additional involvement tasks identified by the research team that require patient and public involvement. A key element of the patient and public involvement coordinator's work is the on-going monitoring of the support requirements of each RUG member. During the introductory meetings the patient and public involvement coordinators completed a support and learning needs form [see Additional file 1] with individual RUG members to understand the different support and learning needs within the group. Support and learning needs are continually revised by the patient and public involvement coordinators by telephone conversations 2 weeks before the RUG meeting. Telephone contact was also preferred by those with hearing problems as RUG members with hearing problems had amplified telephones at home. Regular contact with the RUG members helps the patient and public involvement coordinator to be aware of RUG members' changing needs so that appropriate support can be provided to facilitate involvement. Patient and public involvement coordinators have links with local support services (such as Admiral Nurses and the Alzheimer's

For peer review only

For peer review only - http://bmjopen.bmj.com/site/about/guidelines.xhtml

Society in the UK) that can provide RUG members with additional support if needed. Patient and public involvement coordinators used information from the support and learning needs forms to put in place individual support arrangements to facilitate each person's involvement. For example, for those with vision problems, patient and public involvement coordinators position themselves close to the person and keep still while talking. People with vision problems are provided with training and RUG materials in large font black print on yellow paper. Patient and public involvement coordinators also use verbal cues to direct people's attention to specific written materials, for example "Look at the first paragraph on page 2 of the hand-out" rather than saying "Look at the hand-out".

Aims and objectives of the study

This study aims to evaluate i) the acceptability and perceived outcomes of Research Awareness Training that was provided to support the involvement of older people with dementia and age-related hearing and/or vision impairment in the research and ii) the perceived impacts of the involvement of older people with dementia and age-related hearing and/or vision impairment on the research from the point of view of RUG members and researchers (Study time frame: Table 1).

Table 1

Study time frame for Manchester, Nicosia, Nice and Athens sites

	Jan 2017	April 2017	Jul 2017	Oct 2017	Jan 2018	April 2018	Oct 2018	Jan 2019
SENSE-Cog project timeline (project month)	13	16	19	22	25	28	34	37
Delivery of Research Awareness Training								
TARS questionnaires								
Semi structured interviews with RUG members and researchers								
Focus group interviews								

For peer review only - http://bmjopen.bmj.com/site/about/guidelines.xhtml

Evaluating acceptability and perceived outcomes of Research Awareness Training

Sample

All RUG members involved in the Research Awareness Training in Manchester, Nicosia, Nice and Athens will be invited to take part in the evaluation of the training. The evaluation will be completed by up to 10 (people) RUG members in each study site (Manchester, Nicosia, Nice and Athens).

Research Awareness Training

The Research Awareness Training (Figure 2) was initially developed as part of the Enhancing the Quality of User Involved Care Planning (EQUIP) [23] programme to give UK National Health Service mental health service users and caregivers an understanding of research and research terminology to support them in working as co-researchers on a mental health research project. EQUIP training involved a 6-day research methods course. However we adapted the training to 6 one hour sessions focussing on key research concepts that were relevant for SENSE-Cog. A shorter version of Research Awareness Training than the original 6 day program was developed following discussion with RUG members in each study site during the introductory meetings. RUG members' preference was for shorter, bite-sized training delivered as needed. Shorter, bite-sized training also facilitated participation of people with memory difficulties.

Figure 2

Patient and public involvement coordinators for each RUG completed a three day training course in English (Table 2) to orient them to the content of the Research Awareness Training and describe how training should be delivered to RUGs (Table 2). Discussion of the principles of patient and public involvement training and orientation to the SENSE-Cog project was delivered by the Public Programmes Team in Manchester [24]. The Public Programmes Team is a specialist unit advising on and delivering patient and public involvement and engagement in national and international health

For peer review only

For peer review only - http://bmjopen.bmj.com/site/about/guidelines.xhtml

research [24]. Research Awareness Training for patient and public involvement coordinators was delivered by Professor Karina Lovell and colleagues at the University of Manchester based on a 'train the trainer model' [23] to enable the patient and public involvement coordinators to adapt the Research Awareness Training for local implementation appropriate for RUG member's language, hearing and/or vision impairments.

Table 2

Patient and public involvement coordinators training

Day 1 Principles of patient and public involvement	Day 2 Research Awareness Training	Day 3 Research Awareness Training
Overview of SENSE-Cog and work package 5 objectives to enable patient and public involvement coordinators to establish and support local RUG members and orientate the members to the SENSE-Cog programme and work package 5 objectives.	Good facilitation skills and techniques.<br>Managing difficult situations; case scenarios.<br>Research Awareness Training topics.	Research Awareness Training topics continued.<br>Adaptation plans for Research Awareness Training topics, and cultural differences to take into consideration.

Patient and public involvement coordinators deliver Research Awareness Training to RUGs using a two-way flow interactive discussion. Two-way flow interactive discussion involves an exchange of ideas where both facilitator and RUG members are active and can prompt one another in the discussion of a particular research topic. Each Research Awareness Training session lasts around 60 minutes. Each training session is delivered to support and complement RUG members' involvement in specific aspects of SENSE-Cog research on a needs basis. For example, Research Awareness Training on qualitative methods is offered immediately prior to the RUGs reviewing a question related to qualitative aspects of SENSE-Cog research.

Measures

For peer review only - http://bmjopen.bmj.com/site/about/guidelines.xhtml

Quantitative and qualitative methods will be used to evaluate the experience of Research Awareness Training for RUG members.

Training Acceptability Rating Scale (TARS) [25]

The TARS [25] was selected for evaluating the training, as the TARS has previously been used to evaluate Research Awareness Training for patient and public involvement volunteers [26,27]. The TARS is a self-administered paper questionnaire and takes approximately 5-10 minutes to complete. The TARS consists of three parts: the first concentrating on appropriateness and acceptability of the training, the second covering effectiveness and competency of the trainers. The first two parts contain items rated on a four-point Likert scale, ranging from 'Not at all' (score 1) to 'A great deal' (score 4). The third and final part includes three open ended questions for RUG members to identify the most useful elements of the training, suggest changes and add additional comments. The Research Awareness Training materials and TARS was translated into Greek and French using the 'forward-translations and back-translations' procedure [28].

Semi structured interview and focus group interview

RUG members who have completed the Research Awareness Training will be approached to take part in a semi-structured interview [see Additional file 2] or focus group interview [see Additional file 3] to give their impressions of the training overall, indicate what insights, understanding and skills they had acquired and how they have applied the knowledge and skills provided by the training within the SENSE-Cog programme.

Procedure

Training sessions will be carried out approximately every 3 months. There is some flexibility in the scheduling of training delivery according to local requirements. For example, one training session in Cyprus was postponed from July to September due to very hot summer weather. Participants will complete the TARS questionnaire at the end of each training session to ensure that those with cognitive or memory impairment can provide immediate feedback for each training session. The

For peer review only - http://bmjopen.bmj.com/site/about/guidelines.xhtml

semi-structured interview will be conducted once participants have completed all 6 training sessions in order to obtain each RUG members' overall impressions of training.

Perceived impacts of the involvement of older people with dementia on the research from the point of view of RUG members and researchers

Sample

24 one to one interviews with RUG members (6 at each site) and 6 one to one interviews with SENSE-Cog researchers will be conducted across all sites. The final sample size will be determined by data saturation [29,30,31], although we anticipate that a sample of approximately 6 interviews will be required.

Additional focus group interviews to capture RUG members' views on the on-going RAT and their experience as a RUG member and their perceived impact on the research process, will allow us to reflect on our programme's progress towards achieving its objectives and make any changes on an on-going basis, such as change of venue or working approach with the RUG members. We will aim to maximise diversity in the study sample to increase the validity and transferability of research findings to other settings. The sample of RUG members will include (i) men and women ages 65 years and above (ii) older people with dementia (iii) older people with dementia and age-related hearing and/or vision impairment (iv) carers with age related hearing and/ or vision impairment. RUG membership was constrained to include only people over the age of 65 due to the focus on older adults in the SENSE-Cog project. Younger people with dementia and younger carers will not therefore be included in the research evaluation. The sample of researchers will include i) researchers at different levels (i.e. work package leads and research assistants), ii) researchers in different SENSE-Cog work packages (see figure 1) who have worked with the RUGs and iii) different countries.

For peer review only

Measures

One to one semi structured interviews and focus group interviews will be conducted with RUG members by the patient and public involvement coordinators in each site to capture RUG members' impression of the training and of their experience as a RUG member and their perceived impact on the research process. One to one semi structured interviews with SENSE-Cog researchers [see Additional file 4] across all sites will be conducted by the project coordinator based in Manchester to assess SENSE-Cog researchers experience of working with RUGs and the impacts that RUGs have had on the research process.

Procedure

8 focus group interviews will be conducted in total with RUG members (2 focus groups in each RUG site). Focus group interviews will take place at approximately 22 months and 37 months following the commencement of RUG activities. These time points were selected in order to record RUG members' impressions at the approximate mid-point and near the end of involvement in the research to capture people's views and opinions during on-going involvement in research. In addition, we chose to collect focus group data at more than one point due to the age of RUG members and the progressive nature of dementia. RUG participants may drop in or out of involvement if their condition fluctuates or if RUG members can no longer provide on-going informed consent to participate in the research evaluation of their involvement. One to one interviews with RUG members will be conducted at approximately 34 months. Similarly one to one interviews with SENSE-Cog researchers will be conducted at approximately 34 months in order to capture researcher's perceptions of RUG involvement near the end of the research program.

Data Analysis

For peer review only

TARS [25] data will be analysed at group level using SPSS (IBM, Armonk, NY, United States) to generate descriptive statistics. The open ended questions from the TARS will be analysed qualitatively, as for the interview data below.

One to one interviews and focus group interviews will be audio-recorded. Audio-recordings in Nice, Nicosia and Athens will be transcribed verbatim in the local languages and then translated by each local patient and public involvement coordinator. The data from all 4 sites will be analysed by the lead Study Coordinator in Manchester using NVIVO qualitative software (QSR International Pty Ltd, Victoria, Australia). The FRAMEWORK method [32] will be used for data analysis. The FRAMEWORK method allows in-depth analysis of key themes across the whole datasets, as well as between individual accounts; as it uses existing topic guide as a starting point for the framework, whereas other thematic approaches may just generate themes directly from the data. In addition, the framework method allows the summaries of data across each case and theme or subtheme into charts using Excel which makes the data accessible to a wider research team.

Data from interviews from each site will firstly be analysed separately to produce themes that are relevant to each site. Coding that is conceptually and characteristically noteworthy will be highlighted, rather than focusing just on frequency of occurrence [32,33]. After separate analysis of interview data for each site, themes identified in separate analyses for each site will then be evaluated for common themes across all sites, as well as identification of distinct issues between sites. Analysis across sites will involve a systematic approach for the patient and public involvement coordinators in each sites to refine the themes, classify them into categories and draw conclusions about differences between sites.

The format of the Guidance for Reporting Involvement of Patients and the Public (GRIPP2) [34] reporting checklists will be used to report the implementation and evaluation of patient and public involvement in this study and we will share our findings on the evaluation of the Research Awareness Training via peer reviewed publication once it is complete.

For peer review only - http://bmjopen.bmj.com/site/about/guidelines.xhtml

Ethics

The study received ethical approval from Manchester University Research Ethics Committee. Additional ethical approvals were sought and obtained for each study site (Nicosia, Nice, Athens), as relevant to local arrangements.

A key ethical issue when working with people with dementia is informed consent, especially how researchers establish whether people have capacity for informed consent and how any changes in capacity are identified. According to the Mental Capacity Act (2005) [35], people should be assumed to have capacity unless otherwise demonstrated. The capacity of participants with cognitive impairment to consent to participate (provided that they have clear and appropriate study information and enough time to make decisions) will be assessed on an on-going basis. All staff members involved in this research completed training in assessing capacity in research.

To support the informed participation in the research, RUG members provided input into the development of easy access user friendly versions of the participant information sheet and consent form [see Additional file 5]. In cases where RUG members are unable to complete the consent form due to low vision, the consent form is read out verbatim. RUG member may then agree verbally to participate, and the patient and public involvement coordinator signs the consent form on behalf of the RUG member, which is then witnessed and countersigned by another SENSE-Cog project researcher.

The patient and public involvement coordinators at each site contact the RUG members via telephone 2 weeks before each training session to establish their capacity to consent, and their understanding of their involvement in the training and the evaluation study. The patient and public involvement coordinator assesses whether RUG members can retain the information discussed with them over telephone or in person at the time of discussion by asking the RUG member to repeat the information explained to them. Pre-meetings take place before each training session or RUG meeting to give the patient and public involvement coordinator the opportunity to speak with the

For peer review only - http://bmjopen.bmj.com/site/about/guidelines.xhtml

participant again to establish their capacity for consent and involvement. A similar approach will be taken prior to the one to one interviews and focus group interviews with RUG members.

Discussion

Dementia research is an international priority. To ensure the relevance of research outcomes to older people with dementia, it is vital the views of older people with dementia are taken into account when designing research [2,3,6,7,8]. There is a small but growing literature on the methodology and impacts of involving people with dementia in dementia research [7,36]. Previous reviews of PPI in health research have recognised a lack of reporting of the impact and benefits of PPI, and where studies have described the impact of PPI, the quality of the evidence is low [37,39,40]. The lack of good quality research on the impacts and benefits of PPI necessitates the need for evidence-based models of good practice of patient and public involvement in health research in general as well as evidence for the impacts and benefits of involvement [37,38,39,40,41]. The need is particularly acute in the case of involving older people with dementia and age-related hearing and/or vision impairment in health research. Very few studies have described and evaluated patient and public involvement with older people with dementia [7,36,42,43,44,45,46].

In the present study, we made particular methodological choices to facilitate the participation of older people with dementia and age-related hearing and/or vision impairment in the research evaluation of Research Awareness Training and the impact of RUGs on the SENSE-Cog research project. We chose a combination of questionnaires, focus groups and one to one interviews, because people with dementia have previously participated in research to investigate their opinions and feelings through the use of questionnaires, focus groups and one to one interviews [46,47,48,49,50]. We also employed procedures that maximise participation of people with dementia in research [6,51,52,53,54]. For example, questionnaires were completed by RUG members at the end of each training session, minimising the demand on memory to recall past

For peer review only

For peer review only - http://bmjopen.bmj.com/site/about/guidelines.xhtml

experiences with training. Completing the questionnaires after each training session also allowed participants to ask for clarification from the patient and public involvement coordinators if they required it. We took a flexible approach for the first sets of focus group interviews, based on whether or not the participant wanted to contribute in the group discussion on the day, with quiet rooms available for rest breaks.

Similarly one to one interviews enable the interviewer to explore opinions in-depth, pick up on non-verbal interactions, check the comprehension of questions and allow participants to take time to reflect and respond [50,51,53]. We are aware that people with dementia may not be able to participate in interviews of long duration [55]. We therefore have planned to take a flexible approach and conduct interviews based on whether or not the RUG participant wants to complete the interview on the day. We will attempt to minimise the overall time of the interview and/or plan in regular breaks during the interviews with quiet rooms available to offer opportunities for rest breaks. The patient and public involvement coordinators will conduct the one to one interviews, as they have an established relationship with RUG members and are aware of the needs and style of communication for each member. Patient and public involvement coordinators will plan in extra time before and after the interview to help make the participant feel comfortable [6,51].

This protocol sets out to evaluate training to support the involvement of older people with dementia and age-related hearing and/or vision impairment and the impact of their involvement in research. Evaluation of the RAT will help us to understand the acceptability and appropriateness of the training, findings will be published to contribute to advancing the knowledge base of research training methods for involvement in general and for this particular population group in particular. The study will facilitate better understanding of best practice for the involvement of older people with dementia and age-related hearing and/or vision impairment in research.

Abbreviations

For peer review only

RUG – Research User Group

TARS – Training Acceptability Rating Scale

GRIPP – Guidance for Reporting Involvement of Patients and the Public

EQUIP - Enhancing the Quality of User Involved Care Planning

MICRA - Manchester Institute for Collaborative Research on Ageing

Declarations

Ethics approval and consent to participate

University of Manchester's Research Ethics Committee 2 approved the study number UREC: 2017-0627-2142

Authors' contributions

IL, PD, BS and SP contributed to the conception and design of the study. JM, PD, IL and SP designed the study protocol. PD and SP critically reviewed the protocol. JM wrote the first draft and was involved in the editing of the manuscript, which was reviewed by PD, BS, IL and SP. All authors read and approved the final manuscript.

Acknowledgements

The authors would like to acknowledge the older people working with us in the UK, France, Cyprus and Greece sites and SENSE-Cog colleagues in these countries coordinating the RUGs. The authors would like to acknowledge Valerie Booth (Manchester RUG member) contribution on reviewing the Plain English Summary section.

Funding

This protocol paper is part of Work Package 5 of the SENSE-Cog project, which has received funding from the European Union's Horizon 2020 research and innovation programme under grant

For peer review only

For peer review only - http://bmjopen.bmj.com/site/about/guidelines.xhtml

agreement No. 668648. The funding body has no part in study design, data collection, data analysis, data interpretation, or manuscript writing

Competing interests

The authors declare that they have no competing interests

Consent for publication

Not applicable

Availability of data and material

Data sharing is not applicable to this article as no datasets were generated or analysed during the current study.

References

1. Alzheimers' Research UK. https://www.dementiastatistics.org/statistics/global-prevalence (2016) Accessed 28th March 2018

2. Gove D, Diaz-Ponce A, Georges J, Moniz-Cook E, Mountain G, Chattat R, Øksnebjerg L; European Working Group of People with Dementia. Alzheimer Europe's position on involving people with dementia in research through PPI (patient and public involvement). Aging Mental Health. 2018;22 (6):723-729 https://doi.org/10.1080/13607863.2017.1317334.

3. Meiland F et al. Technologies to Support Community-Dwelling Persons With Dementia: A Position Paper on Issues Regarding Development, Usability, Effectiveness and Cost-Effectiveness, Deployment, and Ethics. JMIR Rehabil Assist Technol. 2017;4(1): doi: 10.2196/rehab.6376.

For peer review only - http://bmjopen.bmj.com/site/about/guidelines.xhtml

4. Dewing J. From ritual to relationship: a person-centred approach to consent in qualitative research with older people who have a dementia. Dementia. 2002;1:157–171

5. Hubbard G, Downs M, Tester S. Including older people with dementia in research: challenges and strategies. Aging & Mental Health. 2003;7:351–362

6. Murphy K, Jordan F, Hunter A, Cooney A, Casey. Articulating the strategies for maximising the inclusion of people with dementia in qualitative research studies. Dementia (London). 2015;14(6):800-24. doi: 10.1177/1471301213512489.

7. Morgan N, Grinbergs-Saull A, Murray M. We can make our research meaningful'. The impact of the Alzheimer's Society Research. 2018. https://www.alzheimers.org.uk/sites/default/files/201804/Research%20Network%20Report%20low-res.pdf. Accessed 23rd September 2018

8. INVOLVE. Briefing notes for researchers. INVOLVE. Eastleigh. UK. http://www.invo.org.uk/wp-content/uploads/2012/04/INVOLVEBriefingNotesApr2012.pdf (2012) Accessed 4th March 2018

9. Going the Extra Mile a strategic review of public involvement in the National Institute for Health Research. https://www.nihr.ac.uk/patients-and-public/documents/Going-the-Extra-Mile.pdf (2015) Accessed 4th March 2018

10. Alzheimer's Society. Outcomes of the James Lind Alliance. Dementia priority setting partnership. 2013.

For peer review only - http://bmjopen.bmj.com/site/about/guidelines.xhtml

https://www.alzheimers.org.uk/download/downloads/id/2226/outcomes_from_the_james_lind_alliance_priority_setting_partnership.pdf Accessed 11th March 2018

11. Deane K, Flaherty H, Daley DJ, et al. Priority setting partnership to identify the top 10 research priorities for the management of Parkinson's disease. BMJ Open. 2014;4(12) https:// doi:10.1136/bmjopen-2014-006434

12. Mockford C, Murray M, Seers K, Oyebode J, Grant R, Boex S, Staniszewska S, Diment Y, Leach J, Sharma U, Clarke R, Suleman R. A SHARED study: the benefits and costs of setting up a health research study involving lay co-researchers and how we overcame the challenges. Research Involvement and Engagement. 2016;2(8): https://doi.org/10.1186/s40900-016-0021-3

13. Poland F, Mapes S, Pinnock H, Katona C, Sorensen S, Fox C, Maidment I D. Perspectives of carers on medication management in dementia: lessons from collaboratively developing a research proposal. BMC research notes. 2014;7:463-463. https://doi.org/10.1186/1756-0500-7-463

14. Paterson C, Allen J, Browning M, Gillian B, Ewings P. A pilot study of therapeutic massage for people with Parkinson's disease: the added value of user involvement Complementary Therapies in Clinical Practice. 2005;11(3):161-171. https://doi.org/10.1016/j.ctcp.2004.12.008

15. Yates LA, Orrell M, Spector A, Orgeta V. Service users' involvement in the development of individual Cognitive Stimulation Therapy (iCST) for dementia: a qualitative study. BMC Geriatrics. 2015;15(4):1-10 doi: 10.1186/s12877-015-0004-5

16. Bunn F, Sworn K, Brayne, C., Iliffe S, Robinson L, Goodman C. Contextualizing the findings of a systematic review on patient and carer experiences of dementia diagnosis and treatment: A

For peer review only

For peer review only - http://bmjopen.bmj.com/site/about/guidelines.xhtml

qualitative study. Health Expectations: An International Journal of Public Participation in Health Care & Health Policy. 2015;18(5):740-753 http://dx.doi.org/10.1111/hex.12162

17. Martin S, Fleming J, Cullum S, Dening T, Rait G, Fox C, Katona C, Brayne C, Lafortune L. Exploring attitudes and preferences for dementia screening in Britain: contributions from carers and the general public. BMC Geriatrics. 2015;15(110) doi: 10.1186/s12877-015-0100-6

18. Brooks J, Savitch N, Gridley K. Removing the 'gag' : Involving people with dementia in research as advisers and participants. Social Research Practice. The SRA journal for methods in applied social research. 2017; 3: 3-14

19. Stevenson M, Taylor B. Involving individuals with dementia as co-researchers in analysis of findings from a qualitative study, Dementia. 2017:1–12. https://doi.org/10.1177/1471301217690904

20. Littlechild R, Tanner D, Hall K. Co-research with older people: Perspectives on impact. Qualitative Social Work. 2015;14(1):18 – 35 https://doi.org/10.1177/1473325014556791

21. Hanson E, Magnusson L, Arvidsson H, Claesson A, Keady J, Nolan M. Working together with persons with early stage dementia and their family members to design a user-friendly technology-based support service. Dementia: The International Journal of Social Research and Practice. 2007; 6(3):411-434. https://doi.org/10.1177/1471301207081572

22. SENSE-Cog. https://www.sense-cog.eu/ (2016) Accessed 26th March 2018

For peer review only - http://bmjopen.bmj.com/site/about/guidelines.xhtml

23. Enhancing the Quality of User Involved Care Planning in Mental Health Services (EQUIP) (2012) http://research.bmh.manchester.ac.uk/equip. Accessed 2nd April 2018

24. Public Programmes Team. https://research.cmft.nhs.uk/facilities-services/public-programmes. (2018) Accessed 2nd April 2018

25. Milne D, Noone S. Teaching and training for non-teachers. The British Psychological Society. Leicester. 1996.

26. Grundy A, Walker L, Meade O, Fraser C, Cree L, Bee P, Lovell K, Callaghan P. Evaluation of a co-delivered training package for community mental health professionals on service user- and carer-involved care planning. Journal of Psychiatry and Mental Health Nursing. 2017;24(6):358–366. doi: 10.1111/jpm.12378

27. Carpenter J, Milne D, Lombardo C, Dickinson C. Process and outcomes of training in psychosocial interventions in mental health: A stepwise approach to evaluation. Journal of Mental Health. 2007; 16(4):505-520. https://doi.org/10.1080/09638230701482329

28. WHO. Process of translation and adaptation of instruments. 2018 http://www.who.int/substance_abuse/research_tools/translation/en/ Accessed 28th September 2018

29. Guest G, Bunce A, Johnson L. How Many Interviews Are Enough? An Experiment with Data Saturation and Variability. Family Health International. 2006;18(1) doi:10.1177/1525822X05279903

For peer review only - http://bmjopen.bmj.com/site/about/guidelines.xhtml

30. O'Reilly M, Parker N. Unsatisfactory saturation: A critical exploration of the notion of saturated sample sizes in qualitative research. Qualitative Research Journal. 2012;1(8) doi:10.1177/1468794112446106

31. Using Excel for Qualitative Data Analysis. http://www.academia.edu/5062702/Using_Excel_for_Qualitative_Data_Analysis. (2018) Accessed 30 April 2018

32. Ritchie J, Lewis J. Qualitative research practice: a guide for social science students and researchers. 2003. London: Sage

33. Braun V. Clarke V. Using thematic analysis in psychology. Qualitative Research in Psychology. 2006;3(2):77-101. http://eprints.uwe.ac.uk/11735

34. Staniszewska S, et al. GRIPP2 reporting checklists: tools to improve reporting of patient and public involvement in research. BMJ. 2017; 358 https://doi.org/10.1136/bmj.j3453

35. Mental capacity Act 2005: http://www.justice.gov.uk/downloads/protecting-the-vulnerable/mca/mca-code-practice-0509.pdf (2013) Accessed 5th March 2018

36. Miah J, Dawes P, Leroi I, Starling B, Edwards S, Parsons S. Types and impact of patient and public involvement in dementia research and clinical service development in Europe: A systematic literature review. 2018. *In press*

For peer review only - http://bmjopen.bmj.com/site/about/guidelines.xhtml

37. Brett J, Staniszewska S, Mockford C, Herron-Marx S, Hughes J, Tysall C, Suleman R. Mapping the impact of patient and public involvement on health and social care research: a systematic review. Health Expectations. 2014;17 (5):637–50. doi: 10.1111/j.1369-7625.2012.00795.x.

38. Domecq JP, Prutsky G, Elariyah T, Wang Z, Nabhan M, Shippee N, et al. Patient engagement in research: a systematic review. Health Services Research. BMC. 2014;14:89. https://doi.org/10.1186/1472-6963-14-89

39. Staley K. Exploring Impact: Public involvement in NHS, public health and social care research. Eastleigh: INVOLVE. http://www.invo.org.uk/wpcontent/uploads/2011/11/Involve_Exploring_Impactfinal28.10.09.pdf. (2009) Accessed 28th March 2018

40. Telford R, Boote J, Cooper C. What does it mean to involve consumers successfully in NHS research? A consensus study. Helath Expectations. 2004. 7(3): 209-220. https://doi.org/10.1111/j.1369-7625.2004.00278.x

41. Staniszewska S, Adebajo A, Barber R, Beresford P, Brady L-M, Brett J et al. Developing the evidence base of patient and public involvement in health and social care research: the case for measuring impact. International Journal of Consumer Studies. 2011;35(6):628-632

42. Hanson E, Magnusson L, Arvidsson H, Claesson A, Keady J, Nolan M. Working together with persons with early stage dementia and their family members to design a user-friendly technology-based support service. Dementia: The International Journal of Social Research and Practice. 2007; 6(3): 411-434. https://doi.org/10.1177/1471301207081572

For peer review only - http://bmjopen.bmj.com/site/about/guidelines.xhtml

43. Littlechild R, Tanner D, Hall K. Co-research with older people: Perspectives on impact. Qualitative Social Work. 2015;14(1):18-35. https://doi.org/10.1177/1473325014556791

44. Stevenson M, Taylor B. Involving individuals with dementia as co-researchers in analysis of findings from a qualitative study, Dementia. 2017:1-12 https://doi.org/10.1177/1471301217690904

45. Tooke J. "Involving people with dementia in the work of an organisation: Service User Review Panels". Quality in Ageing and People. 2013;14(1):56-65, https://doi.org/10.1108/14717791311311111

46. Tanner D. Co-research with older people with dementia: experience and reflections. Journal of Mental Health. 2012;21(3):296–306

47. Cheston R, Bender M, Byatt S. Involving people who have dementia in the evaluation of services: A review. Journal of Mental Health. 2000;9(5):471-479 https://doi.org/10.1080/09638230020005200

48. Acton GJ, Mayhew PA, Hopkins BA, Yauk S. Communicating with individuals with dementia: The impaired person's perspective. Journal of Gerontological Nursing. 1999;25:6–13

49. McKeown J, Clarke A, Ingleton C, Repper J. Actively involving people with dementia in qualitative research. Journal of Clincal Nursing. 2010; 19(13-14):1935-43. doi: 10.1111/j.1365-2702.2009.03136.x.

50. Clare L, Rowlands J, Bruce E, Surr C, Downs M. The Experience of Living with Dementia in Residential Care: An Interpretative Phenomenological Analysis. The Gerontologist:2008;48(6)

For peer review only - http://bmjopen.bmj.com/site/about/guidelines.xhtml

51. Hellstrom I, Nolan M, Nordenfelt L, Lundh U. Ethical and methodological issues in interviewing persons with dementia. Nursing Ethics. 2007; 14:608–619

52. Downs M. The emergence of the person in dementia research. Ageing and Society. 1997;17:597–607

53. Nygard L. How can we get access to the experiences of people with dementia? Scandinavian Journal of Occupational Therapy. 2006:13;101–112

54. Kitwood T. Dementia Reconsidered: The Person Comes First (Rethinking Ageing). Open University Press, Maidenhead. 1997

55. Bartlett R. Modifying the diary interview method to research the lives of people with dementia. Qualitative health research. 2012;22(12):1717-1726

List of Figures

Figure 1

Title: SENSE-Cog work packages

Legend: SENSE-Cog aims to investigate the combined impact of dementia and age-related hearing and/or vision impairment and to develop new tools to improve quality of life for older Europeans. The project started in January 2016 and will run until December 2020. Involvement of older people with dementia in SENSE-Cog is a significant part of the overall work programme.

For peer review only

For peer review only - http://bmjopen.bmj.com/site/about/guidelines.xhtml

665

666 Figure 2

667 Title: Adapted Research Awareness Training

668 Legend: The adapted Research Awareness Training for RUG members consists of 6 one hour sessions focussing on key research concepts that are relevant for SENSE-Cog. The training was developed following discussion with RUG members in each study site. RUG members' preference was for shorter, bite-sized training delivered as needed.

672

673

674

675 List of additional files:

676

677 Additional file 1

678 File format: Word

679 Title of data: Support and Learning Needs Form

680 Description of data: Support and Learning Needs Form used during introductory meeting with RUG members to establish individual needs/preferences for resources during RUG meeting and training sessions

683

684

685 Additional file 2

686 File format: Word

687 Title of data: RUG Members Interview Topic guide

688 Description of data: RUG one to one semi structured interview guide

689

690 Additional file 3

For peer review only - http://bmjopen.bmj.com/site/about/guidelines.xhtml

File format: Word

Title of data: RUG Members Focus Groups Interview guide

Description of data: Focus group interview guide

Additional file 4

File format: Word

Title of data: SENSE-Cog Researchers Interview Guide

Description of data: One to one semi structured interview guide

Additional file 5

File format: Word

Title of data: Easy access participant information sheet and consent form

Description of data: Examples of the easy access participant information sheet and consent form used with SENSE-Cog RUGs to ensure informed participation in the evaluation study.

For peer review only - http://bmjopen.bmj.com/site/about/guidelines.xhtml

Supplementary file 3 – PPI activity Monitoring Form

**RUG Site**	
Work Package	
Project lead/ Researcher	
Dates of Involvement	
Work identified	
PPI Input	
Feedback from RUG	
Action/s taken as a result of the feedback:	
Action not taken with a reason:	
Any other comments from Researcher / Project lead	

For peer review only

Supplementary file 4 – RUG Semi structured interview topic guide

*Questions to be paraphrased by patient and public involvement coordinators

1. What did you think about the Research Awareness Training?

2. Do you feel the Research Awareness Training sessions helped you in your role as a member of the Research User Group?

3. Did you feel that your thoughts / input were listened to and valued?

4. Did you feel that your thoughts / input where useful to the SENSE-Cog research?

5. Were you given feedback from SENSE-Cog researchers / coordinators on where the Research User Group member's had had an impact?

6. Do you feel your experience of being a Research User Group member matched up to how the role was originally described to you? Please explain:

7. To what extent do you feel you were able to contribute to the involvement tasks relating to the SENSE-Cog programme?

8. To what extent do you feel your involvement:

- Impacted on the different tasks within SENSE-Cog?
- Will impact on for the end users (people with dementia and age-related hearing and/or vision impairment)?

For peer review only

9. In terms of your role as a Research User Group member within SENSE-Cog, to what extent do you feel you were:

a) Valued as a partner in this process?

b) Supported to get involved in the different tasks and opportunities within SENSE-Cog?

10. Thinking about your involvement in the different tasks can you talk a bit about your relationship with:

a) The researchers, how they supported you and communicated with you?

b) The Research User Group coordinators, how they supported you and communicated with you?

For peer review only

Supplementary file 5 – Researchers interview topic guide

1. What is your role within the SENSE-Cog Programme?

2. Which work package/s are you involved in?

3. What are your views on involving Research User Groups?

4. Have you been involved in any previous work involving Research User Groups?

5. What has been you experience of involving Research User Groups in the SENSE-Cog research programme:

   a) When were they involved?

   b) What were they involved in? and how did this help?

   c) Did you feel supported in involving Research User Groups?

   d) Did you feel confident in involving Research User Groups?

6. In what ways do you think the involvement of Research User Groups has impacted on your work in the SENSE-Cog programme?

7. Do you think you will be involving Research User Groups in other areas of your future work programmes?

8. Any other comments?

For peer review only - http://bmjopen.bmj.com/site/about/guidelines.xhtml

For peer review only

BMJ Open

The impact of involving people with dementia and their care partners in research: A qualitative study

Journal:	*BMJ Open*
Manuscript ID	bmjopen-2020-039321.R1
Article Type:	Original research
Date Submitted by the Author:	31-Aug-2020
Complete List of Authors:	Miah, Jahanara; University of Manchester, Division of Neuroscience and Experimental Psychology; Manchester University NHS Foundation Trust, Public Programmes Team<br>Parsons, Suzanne; Manchester University NHS Foundation Trust, Public Programmes Team<br>Lovell, Karina; The University of Manchester, The School of Nursing, Midwifery and Social Work<br>Starling, Bella; Manchester University NHS Foundation Trust, Public Programmes Team<br>Leroi, Iracema; Global Brain Health Institute, School of Medicine; University of Manchester, Division of Neuroscience and Experimental Psychology<br>Dawes, Piers; University of Manchester, Manchester Centre for Audiology and Deafness (ManCAD); Macquarie University, Department of Linguistics, Australian Hearing Hub
**Primary Subject Heading**:	Neurology
Secondary Subject Heading:	Evidence based practice, Geriatric medicine, Qualitative research, Research methods, Patient-centred medicine
Keywords:	QUALITATIVE RESEARCH, Dementia < NEUROLOGY, PUBLIC HEALTH

SCHOLARONE™
Manuscripts

I, the Submitting Author has the right to grant and does grant on behalf of all authors of the Work (as defined in the below author licence), an exclusive licence and/or a non-exclusive licence for contributions from authors who are: i) UK Crown employees; ii) where BMJ has agreed a CC-BY licence shall apply, and/or iii) in accordance with the terms applicable for US Federal Government officers or employees acting as part of their official duties; on a worldwide, perpetual, irrevocable, royalty-free basis to BMJ Publishing Group Ltd ("BMJ") its licensees and where the relevant Journal is co-owned by BMJ to the co-owners of the Journal, to publish the Work in this journal and any other BMJ products and to exploit all rights, as set out in our licence.

The Submitting Author accepts and understands that any supply made under these terms is made by BMJ to the Submitting Author unless you are acting as an employee on behalf of your employer or a postgraduate student of an affiliated institution which is paying any applicable article publishing charge ("APC") for Open Access articles. Where the Submitting Author wishes to make the Work available on an Open Access basis (and intends to pay the relevant APC), the terms of reuse of such Open Access shall be governed by a Creative Commons licence – details of these licences and which Creative Commons licence will apply to this Work are set out in our licence referred to above.

Other than as permitted in any relevant BMJ Author's Self Archiving Policies, I confirm this Work has not been accepted for publication elsewhere, is not being considered for publication elsewhere and does not duplicate material already published. I confirm all authors consent to publication of this Work and authorise the granting of this licence.

For peer review only

Title page: The impact of involving people with dementia and their care partners in research: A qualitative study

Jahanara Miah[1,2] Jahanara.miah@manchester.ac.uk

Suzanne Parsons[2] Suzanne.Parsons@mft.nhs.uk

Karina Lovell[3] Karina.lovell@manchester.ac.uk

Bella Starling[2] Bella.Starling@mft.nhs.uk

Iracema Leroi[1,4] Iracema.leroi@tcd.ie

Piers Dawes[5,6] Piers.dawes@mq.edu.au *corresponding author

[1]University of Manchester, Division of Neuroscience and Experimental Psychology, Manchester, UK

[2]Manchester University NHS Foundation Trust, Public Programmes Team, Manchester, UK

[3]University of Manchester, The School of Nursing, Midwifery and Social Work, Manchester, UK

[4]Global Brain Health Institute, School of Medicine, Dublin, IE

[5]University of Manchester, Manchester Centre for Audiology and Deafness (ManCAD), Manchester, UK

[6]Macquarie University, Department of Linguistics, Australian Hearing Hub, Sydney, NSW, AUS

Keywords: Patient and Public Involvement, Co-production, Dementia, Impact, Evaluation, Europe

Word count: 3474

For peer review only - http://bmjopen.bmj.com/site/about/guidelines.xhtml

The impact of involving people with dementia and their care partners in research: A qualitative study

Abstract

Objectives: We aimed to evaluate the impact of Patient and Public Involvement (PPI) at each stage of the research cycle in a dementia research programme.

Design: We used monitoring forms to record the impact of the research programme's PPI at different stages of research and qualitative interviews with all participants to evaluate the impact of PPI.

Setting: We evaluated Research User Groups (RUGs - older people with dementia and care partners) which were established to provide PPI support for the research programme in multiple European sites.

Participants: We purposively sampled RUG members (n=34) and researchers (n=13) who had participated in PPI activities. Inclusion criteria for the study were: (a) RUG members who had participated in the Research awareness training (RAT) and in PPI activities and had the capacity to consent; (b) researchers who involved RUGs in their work.

Results: *Impact on the research:* Changes to the study conduct were made as a result of the feedback from RUGs. These included prioritisation of clinical recommendations, the wording of study information and recruitment materials, the content and layout of the user interface for a computerised memory test, interpretation of intervention results and advice on dissemination avenues. *Impact on RUG members*: They reported that involvement had given them a sense of purpose and satisfaction. Their perception of health research changed from being an exclusive activity to one, which lay people, could have meaningful involvement. *Impact on researchers*: PPI was a new way of working and interacting with PPI members had given them insight into the impact of their work on people living with dementia.

Conclusions: PPI can have a substantial impact on dementia research and the people involved in the research. To justify the time and expense of PPI, the advantageous practical impacts of PPI should be systematically recorded and consistently reported.

Strengths and limitations of this study

- PPI monitoring forms allowed systematic monitoring and evidencing demonstrable impact.
- RUGs had limited control over what level or type of PPI activities they were tasked with.

For peer review only - http://bmjopen.bmj.com/site/about/guidelines.xhtml

- We interviewed researchers involved in a project that contained a strong element of PPI.
- Interviews conducted by PPI coordinators may have biased RUG participant responses.
- The RUG could have included people from more diverse backgrounds.

Introduction

Recognition that considering the views of end-users in health research improves the relevance and quality of health research has raised the profile of patient and public involvement (PPI).[1-13] PPI reflects the growing democratisation of health research and the importance of research impact. PPI ensures that research questions, conduct and outcomes are relevant and meaningful to patients and the public.[11,14-18]

In countries such as the UK, PPI is required by many funders,[19] as well as an ethical obligation.[20] Researchers are encouraged to involve patients and the public at all stages of the research cycle.[21] However, substantial challenges with the conception and implementation of PPI are still evident.[22-25] Although many health research studies acknowledge the beneficial impacts of PPI[23], various reviews highlight that the reporting of PPI is often of poor quality and evidence of impact is weak.[23-28] Thus, there is a clear need to demonstrate the impact of PPI on the research process more rigorously. Detailing the contribution made by PPI enables learning how to do PPI effectively and how to identify key indicators of impact.[29,30] In the context of the international multi-site dementia research programme, SENSE-Cog (www.sense-cog.eu), our aims were to: (i) identify the impact of PPI at each stage of the research cycle[31]; (ii) provide a model for recording the impacts of PPI; and (iii) explore the experiences and perceptions of people with dementia, their care partners and dementia researchers of PPI in the research programme.

Context of the SENSE-Cog Patient and Public Involvement work

SENSE-Cog,[32] is a five-year (2016-2020) European research programme investigating the combined impact of ageing-related hearing and vision impairment on mental well-being. It involves the exploration of longitudinal datasets to understand the interplay of hearing, vision and cognitive impairment,[33-35] the validation of assessments adapted for people with dementia and hearing and/or vision impairment,[36] and the evaluation of a home-based hearing and vision support intervention in improving quality of life for people with dementia.[37,38,39] As part of this programme, a dedicated work package on PPI[40] was embedded as a cross cutting theme (Figure 1) to involve older people with

For peer review only - http://bmjopen.bmj.com/site/about/guidelines.xhtml

dementia and care partners. This involved establishing Research User Groups (RUGs) in Manchester (UK), Nice (France), Nicosia (Cyprus) and Athens (Greece) for PPI at each stage of the SENSE-Cog programme. Each RUG consisted of seven to nine RUG members, who were people with dementia and their care partners. RUG members were provided with research awareness training (RAT; outlined elsewhere [39]) to equip them with the skills and knowledge to guide their involvement in the research and to enable them to contribute to the research programme. Each RUG site was managed and supported by a local PPI coordinator in each of the four sites.[40] We reported all features of the PPI process according to the GRIPP2 short form recommendations[30] (Supplementary file 1) in this paper, and the full protocol involving the RUGs is published elsewhere.[40]

Figure 1:

The published protocol[40] outlining the PPI activities pertaining to the entire SENSE-Cog programme, was established by the RUGs at the outset of the research programme. Additional PPI activities were added at different stages on an 'as needed' basis by local RUGs and to include local contextualised content for the PPI activities. These activities were coordinated both centrally by the lead PPI coordinator based in Manchester, as well as locally by the site-specific coordinators. For the basic programme-wide PPI activities, the central PPI coordination team, RUG and cross-site researchers worked together to agree on the key objectives, formulate the PPI questions, and develop and revise the materials for the PPI activities. The central PPI coordinator supported the local RUGs and their PPI coordinators to implement and shape the activities in a manner that was appropriate and relevant to their local needs.

Methods

Patient and public involvement

A member of the Manchester RUG provided PPI on this paper to ensure it was informed by the perspectives of a person with experience of involvement in health research. PPI input was obtained by a face-to-face consultation. The RUG member first reviewed the structure of the paper, including a rationale, the introduction, background and methods sections. The RUG member suggested the use of diagrams to illustrate the main findings and contributed ideas on how to categorise the findings. We acted on this by using a table to illustrate the key PPI activities in this paper. Second, the RUG

For peer review only - http://bmjopen.bmj.com/site/about/guidelines.xhtml

member reviewed and commented on the draft paper. The RUG member supported the findings and approved the final paper. PPI in this paper provided the perspective of a person with experience of involvement in health research. The RUG member requested not to be named on this paper.

Monitoring and evaluating the impact of the SENSE-Cog PPI programme

We used two methods to monitor and evaluate the PPI work package within SENSE-Cog programme, (a) PPI monitoring forms (Supplementary file 2), throughout the research programme; and (b) qualitative interviews conducted towards the end of the programme.

- *Monitoring process:*

For all PPI activities, local PPI coordinators kept monitoring logs of the feedback from RUG members at each site (Figure 2). Types of feedback sent back to the research teams included: the wording of an adapted rating scale evaluating support care needs; the wording of participant information sheets; content and presentation of the intervention manual; aspects of the intervention itself (a multi-module intervention involving hearing and vision rehabilitation supported by a therapist); adaptation of a hearing aid guide for person with dementia (PwD) for the intervention manual; confirmed the researchers' analysis and refined the coding framework (Table 1). The completed PPI monitoring logs from the sites were sent to the central PPI coordinator in Manchester for recording purposes, and were then forwarded to the relevant work package research team for comments who recorded the actions taken to address the feedback on the monitoring form. All RUGs were given updates on the progress of the overall SENSE-Cog programme every 18 months to inform them of new developments and outputs and for the RUGs to witness how their feedback had shaped different aspects of the work. The PPI monitoring forms were examined by the central PPI coordinator and impacts and justifications for no action following RUG recommendations extracted and itemised in a table format (Table 1).

Figure 2:

- *Qualitative interviews:*

Participants for the qualitative sub-study

Participants for the impact evaluation were purposively sampled from two of the PPI working groups:

For peer review only - http://bmjopen.bmj.com/site/about/guidelines.xhtml

(a) RUG members (n=34) who had participated in the RAT and in PPI activities and had the capacity to consent. Establishing whether potential participants with cognitive impairment can give informed consent to was assessed by staff trained, further details on the assessment of capacity are available elsewhere;[40] (b) Researchers who involved RUGs in their work.

We aimed for a sample size of 6 RUG participants in each RUG site and 6 researchers across all sites to ensure representation and was determined by data saturation, the point when no new information was obtained. We examined the coding framework data, the characteristics of the participant quotes and the findings, and decided to end the at the point when no new information emerged but included three more interviews to check that data saturation has been reached. [41,42] Thirty-four RUG members were invited for interviews. Twenty-three RUG members responded and participated in the interviews. Eighteen researchers were approached for interviews, thirteen researchers responded and participated in the interviews. A total of thirty-six interviews were conducted (Table 2).

Table 2: Characteristics of participants

	**RUG members**				**Researchers**			
**RUG Site**	**Person with dementia**		**Care partner**		**Researcher**		**Researcher project manager**	
	**male**	**female**	**male**	**female**	**male**	**female**	**male**	**female**
Manchester	3	0	0	4	0	5	1	0
Nicosia	2	1	1	1	0	2	0	2
Nice	1	1	0	3	0	1	0	0
Athens	2	1	0	3	0	1	1	0
**Total**	**8**	**3**	**1**	**11**	**0**	**9**	**2**	**2**

Procedure for qualitative sub-study

We wrote two interview schedules, one for RUG participants (Supplementary file 3) and one for researchers (Supplementary file 4). The interview schedule for RUG participants was designed to understand their experience as a RUG member, participating in PPI activities and the perceived impact of their input on the research process. The interview schedule for researcher participants was developed to explore the researcher's view of PPI, their experience of working with RUGs and their perceived impact of PPI on the research.

Interviews with RUG participants, lasting about an hour each, were conducted between October 2018 and October 2019 by local PPI coordinators in each site. The interviews were conducted separately or

For peer review only

For peer review only - http://bmjopen.bmj.com/site/about/guidelines.xhtml

in pairs as preferred by the person with dementia and care partners. Interviews with researcher participants were conducted between January 2019 and September 2019 by the central PPI coordinator (JM) in English, either by phone of face-to-face.

Interviews with English speaking participants were audio-recorded and then transcribed verbatim. Interviews in Greek and French were transcribed verbatim in the local language and translated by the local PPI coordinators. Personal information was removed from transcripts.

Qualitative data analysis

Researcher and RUG data were pooled across sites and analysed. We undertook thematic analysis[43] to draw out the main characteristics of RUG members and researchers experiences. JM undertook the primary analysis, reading transcripts numerous times to develop open codes. Various levels of open coding took place from line-by-line coding. JM and SP independently examined the data to identify themes and reach consensus on interpretation. Themes were then assigned codes according to the Framework method[44] using NVivo software version 11 (QSR International, Doncaster, Australia). This method allows the data to be structured into a chart with columns and rows aiding the identification and grouping of text into relevant parts.

The coding framework for the RUG participants was emailed to local site PPI coordinators who were asked to provide input on a draft of the coding framework. Any additional themes identified by coordinators were added to the list of themes. Datasets were then re-analysed according to the final coding framework. We stopped data collection when no new information was being generated from analysis.[41]

Ethical approval

The study was conducted in Manchester, Nicosia, Nice and Athens. The study received ethical overall approval from Manchester University Research Ethics Committee. Additional ethical approvals were sought and obtained for each study site; in Nicosia approved by the Cyprus National Bioethics Committee, in Nice by “Comité de Protection des personnes Sud Est I”, and in Athens by Ethical Committee of Health Sciences and Scientific Committee of the Eginition Hospital of the National and Kapodistrian University of Athens.

According to the Mental Capacity Act (2005),[45] people should be assumed to have capacity unless otherwise demonstrated. All researchers involved in this research completed training in assessing

For peer review only - http://bmjopen.bmj.com/site/about/guidelines.xhtml

capacity in research. Participants were provided with easy access user-friendly versions of the participant information sheet and consent form and enough time to think about whether to participate in the study. Consent was assessed on an on-going basis. Participants provided informed written consent prior to the interview. Further information on consenting procedure is available elsewhere.[40]

Results

Impact of PPI on research

PPI resulted in specific changes at all stages of the research from identifying and prioritising the research agenda to dissemination (Table 1). In some cases, changes recommended by PPI were not implemented. Reasons that changes could not be implemented included scientific or regulatory requirements (e.g. not being able to make changes to validated psychometric tests, not being able to supply clinical diagnoses on the basis of tests given for a research study, not being able to shorten the participant information sheet due to ethical governance requirements) or limited financial or personnel resources (e.g. being able to produce a study newsletter only four times a year rather than more frequently).

RUG members' perspectives

Four themes were identified: facilitators to contributing, meaningful PPI, personal gains and perceived impact of involvement on research. In the following quotes, the site is reported first (i.e. 'Manchester', 'Nice', 'Nicosia' or 'Athens'). 'RUG (number)' refers to the individual RUG member being quoted. 'PwD' and 'care partner' refers to a person with dementia or a care partner for someone with dementia, respectively.

Facilitators to contributing in PPI

Participants discussed the benefits of group work. Group work was viewed as peer support for participants, which was seen as a valuable way to help understand each other's circumstances and needs to support each other. Participants reported that they had gained new knowledge and discussed the benefits of receiving the RAT for the role, highlighting that the structuring of the training alongside the involvement tasks as factors that helped them to contribute to research. RUG participants also reported that the support provided by the local PPI coordinators made them feel that they were

For peer review only - http://bmjopen.bmj.com/site/about/guidelines.xhtml

231 making a valuable contribution. A participant commented on the importance of the *'human*
232 *relationship' (Athens, RUG4, care partner)* with the coordinator.

For peer review only

For peer review only - http://bmjopen.bmj.com/site/about/guidelines.xhtml

233 Table 1: Summary of key PPI activities and outcomes linking to the stage of the research cycle

PPI activities for RUGs	Outcomes due to PPI input	PPI input not acted on
**Identifying and prioritising the research agenda**		
• International clinical practice recommendations: Recognition and management of hearing and vision impairment in dementia.<br><br>To contribute to development, relevance, and prioritisation the guidelines	-Support for clinical practice recommendations confirmed the relevance of the work.<br><br>-Prioritised recommendations used in the final document e.g. "Train GP's to query if older patients have had their eyes/ ears tested", "The benefits of wearing hearing aids should be highlighted to both PwD and care-partners, "reduce burden due to communication difficulties"<br><br>-Final recommendations endorsed by RUG members.	
**Design**		
• E-checker toolkit<br><br>To contribute to developing an online screening toolkit	-Instructions made clearer.<br>-Text size increased and incorporated throughout the interface for ease of access.<br>-instructions on how to proceed test added.<br>-The layout changed to make it more spontaneous, less formal and more inviting as suggested.<br>-Feedback after each test rather than all at the end.	-The toolkit cannot provide a clinical diagnosis as suggested by PPI because it is an unvalidated test used for research purposes only.

For peer review only - http://bmjopen.bmj.com/site/about/guidelines.xhtml

	-Result feedback format changed.<br>-Female voice included.<br>-Informant questionnaire changed from ‘informant’ to “a person who knows you well”.<br>-Grey boxes changed to a brighter colour.<br>-Cheering for correct answers removed.	
• E-checker toolkit<br><br>User test E-checker. Provide feedback on appearance, usability, the feasibility of the E-checker.	-Font and layout changed to make it easier to see and use.<br>-Instructions simplified.	
• Sensory support Intervention<br><br>To comment on intervention design	-Inclusion criteria changed to include people with more advanced dementia.<br>-The number of visits reduced to minimise the burden on PwD and care partners.<br>-Recruitment team briefed to ensure they are clear to participants about the possible benefits of participating.<br>-Recruitment team briefed to ensure that people randomised to the Care as Usual group know how to access support for their impairment/s.	

For peer review only

• Paper participant Information Sheet (PIS)<br><br>• Audio-visual PIS<br><br>To provide comments on paper and audio-visual PIS	-Language simplified and spelling errors corrected on the paper version.<br>-Supplementary text changed alongside audio-voice on audio-visual PIS.	-Change to shorten the length of the paper participant information sheet could not be made due to ethics requirements.<br>-The voice-over on audio-visual participant information sheet was not changed to female voice due to limited resources.
• Public recruitment poster<br><br>To provide feedback on the presentation, readability, text, images, wording and layout of the poster	-'Dementia research' on title added to encourage/draw attention from target population.<br>-Clarified random allocation, specified as 'group 1 - Care as usual and group 2 – intervention.	

For peer review only

For peer review only - http://bmjopen.bmj.com/site/about/guidelines.xhtml

• Communication guidance<br><br>To advise on contents of communication manual, and materials for use with participants and their study partners	-Communication manual supplemented with tips leaflet from Action on Hearing Loss.<br>-Training delivered to sensory support therapists included advice about adjusting the communication manual to fit with the current abilities/stage of the person with dementia, to maximise their co-operation and inclusion.	-The communication manual was not made into a storyboard format due to limited resources.
• Hearing aid leaflet<br><br>Contribute to developing a dementia-friendly hearing aid user guide	-Changed the leaflet size to A5.<br>-More white space created to make it easier to read.<br>-Font size increased.<br>-Instruction wording changed as suggested throughout the leaflet.<br>-The photo on a front-page removed and changed to hearing aid cartoon image.	
• Glasses care leaflet<br><br>Contribute to developing dementia-friendly glasses care guidance leaflets	-Changed the size, layout, font and wording of the leaflet.<br>-Cross and ticks used to indicate 'do's and don'ts'.<br>-The Sensory Support Therapists (SSTs) to clarify the cloth, wipes and use of glasses straps with the participants during their visits.	

For peer review only - http://bmjopen.bmj.com/site/about/guidelines.xhtml

• Model validation<br><br>Contribute to developing the economic model validation	-Economic model shaped by RUG members experiences<br>-RUG members confirmed research reports concerning health economic impacts of dementia.	
**Data gathering**		
• Sensory Intervention diaries<br><br>To provide feedback on participant and study partners' diary	-Diaries were provided at each visit rather than together as a booklet at the end.	
• Interview questions<br><br>• To advise on qualitative interview schedules on questions for PwD with hearing aids	- Language simplified to ask about people's experiences of using hearing aids.<br>-Confirmed the relevance of the questions.	
• Clinical questionnaires<br><br>To provide feedback on clinical questionnaires	-Spelling errors corrected.	
• RUD-Lite questionnaire<br><br>To advise on the meaning of RUD-lite questionnaire	-Supplementary notes on RUD-Lite questionnaire added to the researcher's manual.	-Questions could not be directly changed on the RUD-lite questionnaire as it is a validated tool for the trial.

For peer review only - http://bmjopen.bmj.com/site/about/guidelines.xhtml

**Analysing & interpreting**		
• Main trial qualitative data interpretation<br><br>Assist with data analysis on selected transcripts to identify codes	- The codes identified by the RUGs confirmed the researchers' analysis and helped to refine the 'coding tree', as well as identify additional themes that were not identified by the researchers.	
• Modelling the impact of sensory interventions on cognitive health	-Discussion of RUG experiences confirmed the research team's interpretation of the results.	
**Dissemination**		
• Dissemination strategy<br><br>Advise on the dissemination plan	-Public/social events incorporated into the dissemination plan.<br>-Local and national stakeholder groups included in the dissemination list for the newsletter and network list.<br>-YouTube videos produced online about current research programme and findings.	
• The impact of sensory interventions on cognitive health<br><br>Advise on dissemination routes for the findings	-Dissemination of findings to a wider public audience via national newspapers, including Daily Mail, The Times.	

For peer review only - http://bmjopen.bmj.com/site/about/guidelines.xhtml

• Nicosia public engagement event<br><br>Provide feedback on content and structure of engagement event	-Changes were made to the presentation agenda to include care partners' perspectives, information, and support for care partners.<br>-2 RUG members gave presentations about their involvement in research.	
• Nice public engagement event<br><br>Provide feedback on content and structure of engagement event	-Wording, layout and language simplified on the presentation slides.<br>- Workshop included an audience discussion on the concept of PPI.	
• Newsletter<br><br>Advise on name of the newsletter, content and format of the project newsletter	-Newsletter named 'SENSE-Cog news'.<br>-Suggestions concerning the content were incorporated to include updates on the studies, ongoing activities within SENSE-Cog with a section on each study site, biographic details of research teams and forthcoming events.<br>-A section on RUGs perspectives included.	-Newsletter published quarterly and not every 2 months as suggested due to limited resources.
• INVOLVE conference 2017<br><br>Provide feedback on a planned conference presentation	-Changes were made to the order of the slides to make it easier to follow.<br>-Additional handout materials included for the presentation to provide more information about the research.	

For peer review only - http://bmjopen.bmj.com/site/about/guidelines.xhtml

• PPI protocol paper<br><br>Contribute to the plain English summary section	-Changes were made to the text to simplify the wording and the approved the plain English summary.	
• PPI scoping review<br><br>Provide feedback on the main findings of the review	-Discussions and recommendations on findings incorporated in the findings section of the paper.	
• AAIC conference poster presentation<br><br>Advise on the contents and presentation of conference paper	-Layout changed on the poster to include more white space to make it visibly appealing, wording simplified, and content re-arranged to make the materials easy to read.<br>-RUGS chose photos of PPI activity to be included on the poster.	
• Age and accessibility workshop<br><br>Provide feedback on a planned workshop presentation	- More details about the support provisions for RUGs were included in the presentation slides.<br>- Wording simplified and texts size increased.<br>-Video of a RUG member talking about her experience of involvement included in the presentation.	
• NIHR PPI and inclusion workshop<br><br>Provide feedback on a planned workshop presentation	- Wording simplified.<br>-Video of a RUG member talking about her experience of involvement included in the presentation.	

For peer review only - http://bmjopen.bmj.com/site/about/guidelines.xhtml

• SENSE-Cog website publicity materials<br><br>Advise on content and images for websites and publicity materials	- Chose photographs for use in the study website and other public-facing materials.<br>-Wording on the website changed to simplify language. The term 'elderly' changed to 'older European citizens' and 'seniors'.	

For peer review only

For peer review only - http://bmjopen.bmj.com/site/about/guidelines.xhtml

Meaningful PPI

Interaction with researchers and having researchers listen to RUG member's opinions to was reported as an important aspect. Receiving feedback was also highlighted as an important factor in letting RUG members know that their contribution was having a tangible impact:

"I feel that our communication was quite satisfactory and apart from that we had feedback. We said something and they 'listened' to it and we move forward, they tell you a little more, something like that….Yes, for sure. Otherwise, I wouldn't be here today. It's a good feeling, to be heard. You gain this feeling that you are not alone and that there are people that care for you, and that what you say can go further" (Athens, RUG5, care partner*)*

Personal benefits of PPI

Face-to-face group meetings provided RUG members the opportunity to share issues with others in similar position. RUG members reported that the experience personally benefited them in terms of gaining new knowledge, interacting with others, giving advice and feeling that they were making a worthwhile contribution. RUG participants felt their involvement exceeded their expectations, due to continuous involvement and training in research awareness:

"Throughout the meetings we felt that what we learned is very important for the lives of individuals. I felt the role was more than was described (role of RUG member), because we participated (learned) in many task trying to find solutions that we have to resolve." (Athens RUG6, PwD)

Perceived impact of involvement on research

RUG members talked about their involvement in dissemination activities, particularly in countries other than the UK. RUG members talked about how providing their perspective provided researchers with a better understanding of the impact of dementia that informed research decisions. RUG members emphasised their contribution in making written materials more accessible for participants and suggesting changes in the interventions and tools developed by the research team. RUG members reported that incremental changes informed by PPI could make a difference to practical outcomes:

For peer review only - http://bmjopen.bmj.com/site/about/guidelines.xhtml

"Well it's just one factor in the complicated chain of conditions that lead to the outcome. So it's not going to have any direct impact on any individual people, but every time a member of the group makes a contribution, it's adding a little bit more information to the whole package of information that's going to improve the lives of the end users in the long term." (Manchester, RUG1, ex-care partner)

Researchers' perspectives

Four themes were identified: limited experience of PPI, acceptance and importance of PPI, beneficial impacts of involvement and challenges. These are presented in table 3, in the quotes, the site is reported first (i.e. 'Manchester', 'Nice', 'Nicosia' or 'Athens') then the researcher number. 'PM' refers to project managers.

Table 3: Researchers' perspectives on impact of involvement

**Themes**	**Item**
Limited experiences of PPI	Of the 13 researchers interviewed, 10 had no previous experience of systematically obtaining patient and public input to planning or conduct of research:<br>*"So it was the first time we've heard about it, on this project. So it took us all a while to understand it, but I mean by going through it and by doing it we understood the importance of it." (Nicosia, R3)*
Acceptance and importance of PPI	There was general acceptance of PPI by researchers and acknowledgement of the importance of the PPI integrated into the research programme. Researchers reported that PPI provided them with the perspective of living with dementia:<br>*"I think it's very useful and I think it gives a perspective to research that we needed to have, because we kind of tend to, you know, sort of take the feeling out of our day to day research and when you have the actual patients explain to them rather than peers, it does give us a different perspective" (Manchester, PM9)*
Beneficial impacts of involvement	Researchers talked about the beneficial impacts of PPI, including refining content and wording of public-facing information and identifying issues not considered by the researchers. One researcher commented that PPI provided insight into what patients and the public think of their work.

For peer review only - http://bmjopen.bmj.com/site/about/guidelines.xhtml

	Researchers valued PPI with respect to understanding the impacts of dementia on daily life and how these impacts should be captured in health economic measurement, ensuring the appropriateness and relevance of health economic measures: *"We were trying to confirm that using dependence as some measure of progression in our economic model would be appropriate and having spoken to the RUG members, they, kind of, confirmed that. And then there were some, kind of, hallmark things that stood out that they all, kind of, agreed on in their discussions.........It has helped in terms of model of validation." (Man, R12)* Some researchers also talked about the emotional impact of listening to RUG members' experiences, which were distressing at times, particularly for researchers not used to patient contact. Researchers reported that their interaction with RUG members led to researchers changing future practice, such as taking patient perspectives into account in planning and prioritising research: *"I've written numerous grant proposals since SENSE-Cog began, and in some of them I've included…wherever it makes sense, I've included a research utilisation for a patient and public involvement group." (Nicosia, PM8)*
Challenges	Researchers talked about challenges, such as PPI suggestions not being able to be implemented because of scientific reasons or resource limitations. Another researcher highlighted the importance of identifying points of research design where PPI would have a critical impact and reported a missed opportunity concerning selection of outcome measures for an intervention study due to time constraints in development of the study protocol.

Discussion:

PPI is proposed as an important means of enhancing the relevance and impact of research.[21,46] However, while the descriptive literature on PPI is growing, there is still little evidence for how PPI actually impacts on research and there has been no clear methodology for recording impact.[22-24,27] Here, we reported a method for capturing the impact of PPI in a multi-centre European research project, outlining the impact of PPI at each stage of the research process. We also provided qualitative

For peer review only

For peer review only - http://bmjopen.bmj.com/site/about/guidelines.xhtml

perspectives of RUG members and researchers concerning their views on the impact of involving people with dementia and care partners in research.

A number of key changes were made to different aspects of the research programme at various points following PPI advice. PPI contributed substantive input to intervention formation, design, recruitment, interpretation of results and dissemination of the research. However, some PPI feedback could not be acted on for the reasons outside the research team's control due to resource restrictions, lack of time or compromising validated psychometric measures. These restrictions on implementing PPI advice are similar to restrictions reported elsewhere.[15,26] Without ongoing advice from the RUGs to the research teams, researchers may not have made appropriate changes on many aspects of the research design to take account of the experience of living with dementia. In addition to concrete changes in the research following RUG input, RUG members reported various personal benefits in contributing to research, including feeling that they were making a useful contribution to research. Researchers reported that the RUG members input gave them an important personal perspective on the relevance and impact of their research for people living with dementia.

Limitations

RUG interview data were collected by the coordinators who were themselves SENSE-Cog project researchers, rather than by an independent researcher. Data collection by SENSE-Cog project researchers may have resulted in responses being overly positive.[43,44]

Although research involvement opportunities for RUGs were advertised via contact with local charities, social organisations and support groups, we received few queries about involvement and no queries from anyone from minority ethnic communities. The RUG groups may not have reflected the diversity within the general community, and this may have limited the perspectives that RUGs were able to offer and of the impact of PPI in this project. Using local community leaders or organisations to access more diverse communities could have increased recruitment from minority groups.[47-49] Future research could explore views of older adults from minority ethnic background and diverse communities in PPI.

For our qualitative work, we only sought the opinions of researchers who were involved in a project that contained a strong element of PPI. The researchers in this project may have been more favourably disposed to PPI than European researchers in general. Another limitation is that RUGs were not involved in the initial conception of the study at the grant application stage. Ideally, PPI should occur from the point of conception of a research programme and around setting research agendas. Due to

For peer review only

For peer review only - http://bmjopen.bmj.com/site/about/guidelines.xhtml

316 time and resource constraints, and possible extra burden we did not involve RUG members in data
317 collection although they were involved in the development of the interview questions.

318 It is appropriate to highlight the issue of the power imbalance[13] between researchers and RUGs. The
319 RUGs had limited control over what level or type of PPI activities they were tasked with in the research
320 programme. In addition, RUG members had limited influence on what feedback were acted on, as
321 they were dependent on researchers listening to their input and acting on their feedback. We
322 provided RUG members with notes of the PPI activities to acknowledge their contribution and
323 provided written responses from the researchers on what actions were taken because of the RUGs
324 contributions.

325 PPI in research has potential benefits, but it does require substantial resources that could otherwise
326 be spent elsewhere. Although this paper highlighted the impact of PPI on the research and
327 documented positive experiences of the RUG members and researchers, we cannot be sure of the
328 impact of these changes on research outcomes (for example, if fewer participants would have been
329 recruited if recruitment materials had not been altered following PPI input). An understanding of the
330 benefit and cost utility of PPI would require a controlled evaluation (for example, recruitment with
331 versus without PPI input on recruitment materials). There is some evidence from controlled
332 evaluations that PPI does have tangible impact: a systematic review on the impact of PPI on enrolment
333 and retention in clinical trials reported that PPI considerably improved the likelihoods of a patient
334 joining a trial compared to recruitment strategies that did not include PPI.[18] However, because
335 recruitment strategies that had used PPI also included non-PPI components, it was difficult to establish
336 the contribution of PPI versus other factors.

337 The overall strength of this study is the demonstrable impacts of PPI on research and the benefits
338 reported for both researchers and RUGs interactions highlight gains in experiential knowledge and
339 learning.

340

341 **Conclusion**

342 This study provides a methodology for recording the impact of PPI in multi-centre health research
343 using systematic ongoing monitoring and feedback throughout the research programme, followed by
344 detailed qualitative exploration of PPI contributors' and researchers' views. To justify the time and
345 expense of PPI, the impacts and benefits of PPI should be systematically recorded and reported for all
346 health research that makes use of PPI.

347 Further information about SENSE-Cog is available at www.sense-cog.eu

For peer review only - http://bmjopen.bmj.com/site/about/guidelines.xhtml

For peer review only

References

1. Tinetti ME, Basch E. Patients' responsibility to participate in decision making and research. *JAMA.* 2013;309:2331–2332.
2. Deane K, Flaherty H, Daley DJ, al e. Priority setting partnership to identify the top 10 research priorities for the management of Parkinson's disease. *BMJ Open.* 2014;4(12).
3. Meiland F, Innes A, Mountain G, et al. Technologies to Support Community-Dwelling Persons With Dementia: A Position Paper on Issues Regarding Development, Usability, Effectiveness and Cost-Effectiveness, Deployment, and Ethics. *JMIR Rehabil Assist Technol.* 2017;4(4).
4. Mockford C, Murray M, Seers K, et al. A SHARED study: the benefits and costs of setting up a health research study involving lay co-researchers and how we overcame the challenges. *Research Involvement and Engagement.* 2016;2(8).
5. Pollard K C, Evans D. *'Theorising service user involvement from a researcher perspective', in Staddon, P. (ed.) Mental Health Service Users in Research.* Bristol,: Policy Press; 2013.
6. Poland F, Mapes S, Pinnock H, et al. Perspectives of carers on medication management in dementia: lessons from collaboratively developing a research proposal. *BMC research notes.* 2014;7:463-463.
7. Paterson C, Allen J, Browning M, Gillian B, P. E. A pilot study of therapeutic massage for people with Parkinson's disease: the added value of user involvement Complementary Therapies in Clinical Practice. *11.* 2005;2:161-171.
8. Brooks J, Savitch N, K. G. Removing the 'gag' : Involving people with dementia in research as advisers and participants. . *Social Research Practice.* 2017;3(14).
9. Stevenson M, Taylor BJ. Involving individuals with dementia as co-researchers in analysis of findings from a qualitative study. *Dementia (London).* 2019;18(2):701-712.
10. Littlechild R, Tanner D, Hall K. Co-research with older people: Perspectives on impact *Qualitative Social Work.* 2015;14(8):18 – 35.
11. Pollard K, Moule P, Evans D, Donald C, AL D, Rice C. User engagement in the co-production of knowledge for Knowledge Exchange in Health and Social Care 2010, Bristol.
12. Tanner D. Co-research with older people with dementia: experience and reflections. *Journal of Mental Health.* 2012;21(3):296–306.
13. Tooke J. "Involving people with dementia in the work of an organisation: Service User Review Panels"

Quality in Ageing and People. 2013;14(1):56-65.

14. Boote J, Wong R, Booth A. Talking the talk or walking the walk? A bibliometric review of the literature on public involvement in health research published between 1995 and 2009. *Health Expect.* 2015;18:44–57.
15. Domecq JP, Prutsky G, Elraiyah T, et al. Patient engagement in research: a systematic review. *BMC Health Serv Res.* 2014.
16. Gamble C, Dudley L, Allam A, et al. Patient and public involvement in the early stages of clinical trial development: a systematic cohort investigation. *BMJ Open.* 2014;4(7).
17. Shippee ND, Domecq Garces JP, Prutsky Lopez GJ, et al. Patient and service user engagement in research: a systematic review and synthesized framework. *Health Expect.* 2015;18(5):1151–1166.
18. Crocker JC, Ricci-Cabello I, Parker A, et al. Impact of patient and public involvement on enrolment and retention in clinical trials: systematic review and meta-analysis. *BMJ.* 2018;28.
19. Department of Health. *Research governance framework for health and social care* London2005.

For peer review only - http://bmjopen.bmj.com/site/about/guidelines.xhtml

20. Iliffe S, McGrath T, Mitchell D. The impact of patient and public involvement in the work of the Dementias & Neurodegenerative Diseases Research Network (DeNDRoN): Case studies. *Health Expectations.* 2013;16(4):351–361.
21. INVOLVE. *Briefing notes for researchers: involving the public in NHS, public health and social care research.* 2012.
22. Staniszewska S, Brett J, Mockford C, Barber R. The GRIPP checklist: strengthening the quality and transparency of reporting for patient and public involvement in research. *Int J Technol Assess Health Care.* 2011;27:391–399.
23. Staniszewska S, Denegri S, Matthews R, V. M. Reviewing progress in public involvement in NIHR research: developing and implementing a new vision for the future. *BMJ Open.* 2018;8.
24. Miah J, Dawes P, Leroi I, Starling B, Edwards S, Parsons S. Patient and public involvement in dementia research in the European Union: a scoping review. *BMC Geriatrics.* 2019;19.
25. Bethell J, Commisso E, Rostad HM, et al. Patient engagement in research related to dementia: A scoping review. *Dementia.* 2018;17(8):944-975.
26. Brett J, Staniszewska S, Mockford C, al. e. Mapping the impact of patient and public involvement on health and social care research: a systematic review. *Health Expectations.* 2014;17(5):637–650.
27. Wilson P, Mathie E, Keenan J, et al. ReseArch with patient and public invOlvement: a RealisT evaluation - the RAPPORT study. *Health Serv Deliv Res.* 2015.
28. Mockford C, Staniszewska S, Griffiths F, Herron-Marx S. The impact of patient and public involvement on UK NHS health care: a systematic review. *Int J Qual Health Care.* 2012;24(1):28–38.
29. Evans D, Coad J, Cottrell K, et al. Public involvement in research: assessing impact through a realist evaluation. *Health Serv Del Res.* 2014.
30. Staniszewska S, Brett J, Simera I, et al. GRIPP2 reporting checklists: tools to improve reporting of patient and public involvement in research. *BMJ.* 2017;358.
31. INVOLVE. Briefing note eight: Ways that people can be involved in the research cycle. https://www.invo.org.uk/posttyperesource/where-and-how-to-involve-in-the-research-cycle/. Accessed 17 Aug 2020.
32. SENSE-Cog project https://www.sense-cog.eu/. Accessed August 17, 2019.
33. Maharani A, Dawes P, Nazroo J, Tampubolon G, Pendleton N, Sense-Cog WP1 group. Visual and hearing impairments are associated with cognitive decline in older people. *Age and ageing.* 2018;47(4):575-581.
34. Maharani A, Dawes P, Nazroo J, Tampubolon G, Pendleton N, SENSE-Cog WP1 group. Cataract surgery and age-related cognitive decline: A 13-year follow-up of the English Longitudinal Study of Ageing. *PloS one.* 2018;13(10).
35. Maharani A, Pendleton N, Leroi I. Hearing Impairment, Loneliness, Social Isolation, and Cognitive Function: Longitudinal Analysis Using English Longitudinal Study on Ageing. *The American journal of geriatric psychiatry.* 2019 27(12):1348-1356.
36. Dawes P, Wolski L, Himmelsbach I, Regan J, Leroi I. Interventions for hearing and vision impairment to improve outcomes for people with dementia: a scoping review. *International psychogeriatrics.* 2019 31:203-221.
37. Hooper E, Simkin Z, Abrams H, et al. Feasibility of an Intervention to Support Hearing and Vision in Dementia: The SENSE-Cog Field Trial. *Journal of the American Geriatrics Society.* 2019 67(7):1472-1477.
38. Regan J, Frison E, Collin F, et al. Individualised sensory intervention to improve quality of life in people with dementia and their companions (SENSE-Cog trial): study protocol for a randomised controlled trial. *Trials.* 2019;20(80).
39. Miah J, Dawes P, Leroi I, Starling B, Lovell K, Parsons S. Evaluation of a research awareness training programme to support research involvement of older people with dementia and their care partners. *Health Expectations.* 2020.

For peer review only - http://bmjopen.bmj.com/site/about/guidelines.xhtml

40. Miah J, Dawes P, Leroi I, Parsons S, Starling B. A protocol to evaluate the impact of involvement of older people with dementia and age-related hearing and/or vision impairment in a multi-site European research study. *Research Involvement and Engagement.* 2018;4.
41. Guest G, Bunce A, Johnson L. How many interviews are enough? An experiment with data saturation and variability. *Field Methods.* 2006;18:59-82.
42. O'Reilly M, Parker N. Unsatisfactory saturation: A critical exploration of the notion of saturated sample sizes in qualitative research. *Qualitative Research Journal.* 2012;1(18).
43. Creswell J, Hanson W, Plano V, Morales A. Qualitative Research Designs Selection and Implementation. . *The Counseling Psychologist.* 2007;35:236-264.
44. Ritchie J, Lewis J. *Qualitative research practice: a guide for social science students and researchers.* . London: Sage; 2003.
45. Mental capacity Act 2005. http://www.justice.gov.uk/downloads/protecting-the-vulnerable/mca/mca-code-practice-0509.pdf Accessed Accessed 5th March 2018.
46. Ocloo J, Matthews R. From tokenism to empowerement: progressing patient and public involvement in healthcare improvement. *BMJ Qual Saf.* 2016;25:626–632.
47. Rojas-García A, Turner S, Pizzo E, Hudson E, Thomas J, R. R. Impact and experiences of delayed discharge: a mixed-studies systematic review. *Health Expect.* 2017;2(1):41–56.
48. Ismail MM, Gerrish K, Naisby A, Salway S, P. C. Engaging minorities in researching sensitive health topics by using a participatory approach. *Nurse Res.* 2014;22(2):44-48.
49. Dudley L, Gamble C, Preston J, et al. What difference does patient and public involvement make and what are its pathways to impact? Qualitative study of patients and researchers from a cohort of randomised clinical trials. *PLoS One.* 2015;10(6).

Acknowledgments

The authors would like to acknowledge the people working with us as part of Research User Groups and SENSE-Cog colleagues coordinating the Research User Groups in the UK, France, Cyprus and Greece.

Funding

This protocol paper is part of Work Package 5 of the SENSE-Cog project, which has received funding from the European Union's Horizon 2020 research and innovation programme under grant agreement No. 668648. Piers Dawes, Suzanne Parsons and Bella Starling are supported by the National Institute for Health Research Manchester Biomedical Research Centre.

Author Contribution

IL, PD, KL, BS contributed to the conception of the study. JM, PD and SP contributed to the design of the study. JM wrote the first draft and was involved in the editing of the manuscript, which was critically reviewed by IL, KL, PD and SP and approved the final manuscript.

For peer review only

For peer review only - http://bmjopen.bmj.com/site/about/guidelines.xhtml

Disclaimer

The funding body has no part in study design, data collection, data analysis, data interpretation, or manuscript writing.

Competing interests

The authors declare that they have no competing interests.

Ethics approval

Ethical Approval was approved by the University of Manchester's Research Ethics Committee UREC: 2017-0627-2142. Additional ethical approvals were sought and obtained for each study site; in Nicosia approved by the Cyprus National Bioethics Committee, in Nice by "Comité de Protection des personnes Sud Est I", and in Athens by Ethical Committee of Health Sciences and Scientific Committee of the Eginition Hospital of the National and Kapodistrian University of Athens.

Data sharing statement

Interviews data from this study are personally identifiable for RUG members and researchers, and are therefore not available for data sharing

Patient consent for publication

Obtained

Figure 1: SENSE-Cog work package aims

Overview of SENSE-Cog research programme investigating the combined impact of ageing-related hearing and vision impairment on mental well-being with a dedicated work package on PPI embedded as a cross-cutting theme.

For peer review only

Figure 2: SENSE-Cog PPI model of working: monitoring and evaluation

PPI model of working in monitoring and evaluation.

For peer review only

For peer review only - http://bmjopen.bmj.com/site/about/guidelines.xhtml

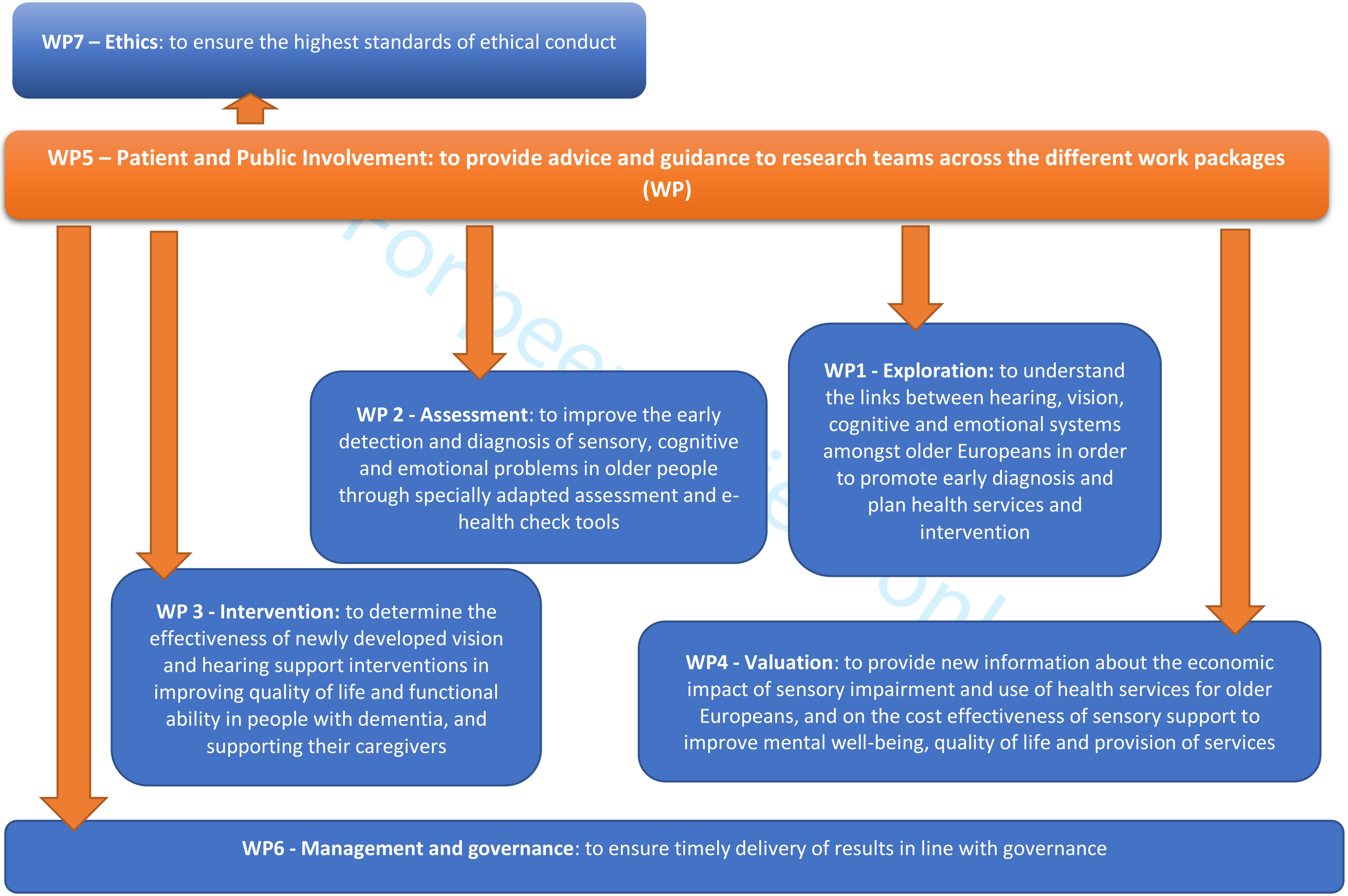
WP7 – Ethics: to ensure the highest standards of ethical conduct
WP5 – Patient and Public Involvement: to provide advice and guidance to research teams across the different work packages (WP)
WP 2 - Assessment: to improve the early detection and diagnosis of sensory, cognitive and emotional problems in older people through specially adapted assessment and e-health check tools
WP1 - Exploration: to understand the links between hearing, vision, cognitive and emotional systems amongst older Europeans in order to promote early diagnosis and plan health services and intervention
WP 3 - Intervention: to determine the effectiveness of newly developed vision and hearing support interventions in improving quality of life and functional ability in people with dementia, and supporting their caregivers
WP4 - Valuation: to provide new information about the economic impact of sensory impairment and use of health services for older Europeans, and on the cost effectiveness of sensory support to improve mental well-being, quality of life and provision of services
WP6 - Management and governance: to ensure timely delivery of results in line with governance

Monitoring

1. PPI activity identified:

- PPI and research team agree on PPI outcomes
- Develop and refine PPI activity and supplementary materials

2. Local PPI coordinators plan PPI activity and facilitation guide:

- Translate materials into Greek and French and plan appropriately to local Research user Groups (RUGs) needs

3. RUG members informed of PPI activity:

- PPI objectives, agenda and activity materials posted to RUG members 2 weeks before meeting
- RUG members confirm attendance for PPI activity or opt out of activity.
- RUG members unable to attend meetings provided feedback via telephone, post or email.

4. During RUG meeting:

- Facilitation of PPI activity led by PPI coordinator or researcher
- PPI coordinator records RUGs feedback on PPI monitoring form

5. Completed monitoring form:

- Copies of PPI monitoring forms forwarded to central PPI team for record keeping.
- PPI monitoring forms forwarded to relevant research team to complete, highlighting actions taken based on RUG feedback and justifications for no actions

6. Feedback to RUG:

- Feedback from research team communicated to RUG members via following RUG meetings, letters and also updated on 18 month progress reports and meetings

Evaluation

Qualitative interviews:

1. RUG members

To understand about their experience as a RUG member, participating in PPI activities and the perceived impact of their input on the research process

2. Researchers

To explore researcher's view of PPI, their experience of working with RUGs and the perceived impact of PPI on the research

Supplementary file 1 – GRIPP2 short form

Section and topic	Item	Reported on page No
1: Aim	Report the aim of PPI in the study	2
2: Methods	Provide a clear description of the methods used for PPI in the study	3,4
3: Study results	Outcomes—Report the results of PPI in the study, including both positive and negative outcomes	6-9
4: Discussion and conclusions	Outcomes—Comment on the extent to which PPI influenced the study overall. Describe positive and negative effects	10,11
5: Reflections/critical perspective	Comment critically on the study, reflecting on the things that went well and those that did not, so others can learn from this experience	10,11

Supplementary file 2 – PPI activity Monitoring Form

**RUG Site**	
Work Package	
Project lead/ Researcher	
Dates of Involvement	
Work identified	
PPI Input	
Feedback from RUG	
Action/s taken as a result of the feedback:	
Action not taken with a reason:	
Any other comments from Researcher / Project Manager	

For peer review only

Supplementary file 3 – RUG Semi structured interview topic guide

*Questions to be paraphrased by patient and public involvement coordinators

1. What did you think about the Research Awareness Training?

2. Do you feel the Research Awareness Training sessions helped you in your role as a member of the Research User Group?

3. Did you feel that your thoughts / input were listened to and valued?

4. Did you feel that your thoughts / input where useful to the SENSE-Cog research?

5. Were you given feedback from SENSE-Cog researchers / coordinators on where the Research User Group member's had had an impact?

6. Do you feel your experience of being a Research User Group member matched up to how the role was originally described to you? Please explain:

7. To what extent do you feel you were able to contribute to the involvement tasks relating to the SENSE-Cog programme?

8. To what extent do you feel your involvement:

· Impacted on the different tasks within SENSE-Cog?

· Will impact on for the end users (people with dementia and age-related hearing and/or vision impairment)?

For peer review only

9. In terms of your role as a Research User Group member within SENSE-Cog, to what extent do you feel you were:

a) Valued as a partner in this process?

b) Supported to get involved in the different tasks and opportunities within SENSE-Cog?

10. Thinking about your involvement in the different tasks can you talk a bit about your relationship with:

a) The researchers, how they supported you and communicated with you?

b) The Research User Group coordinators, how they supported you and communicated with you?

For peer review only

Supplementary file 4 – Researchers interview topic guide

1. What is your role within the SENSE-Cog Programme?

2. Which work package/s are you involved in?

3. What are your views on involving Research User Groups?

4. Have you been involved in any previous work involving Research User Groups?

5. What has been you experience of involving Research User Groups in the SENSE-Cog research programme:

   a) When were they involved?

   b) What were they involved in? and how did this help?

   c) Did you feel supported in involving Research User Groups?

   d) Did you feel confident in involving Research User Groups?

6. In what ways do you think the involvement of Research User Groups has impacted on your work in the SENSE-Cog programme?

7. Do you think you will be involving Research User Groups in other areas of your future work programmes?

8. Any other comments?

For peer review only - http://bmjopen.bmj.com/site/about/guidelines.xhtml

Standards for Reporting Qualitative Research (SRQR)

O'Brien B.C., Harris, I.B., Beckman, T.J., Reed, D.A., & Cook, D.A. (2014). Standards for reporting qualitative research: a synthesis of recommendations. *Academic Medicine, 89(9)*, 1245-1251.

No. Topic	Item
**Title and abstract**	
S1 Title	Line 1 & 2
S2 Abstract	Page 2
**Introduction**	
S3 Problem formulation	Page 2
S4 Purpose or research question	Page 3
**Methods**	
S5 Qualitative approach and research paradigm	Page 5
S6 Researcher characteristics and reflexivity	Page 21 & 22
S7 Context	Page 3 & 4
S8 Sampling strategy	Page 5 & 6
S9 Ethical issues pertaining to human subjects	Page 6 & 7
S10 Data collection methods	Page 5 & 6
S11 Data collection instruments and technologies	Page 5 & 6
S12 Units of study	Page 6
S13 Data processing	Page 7
S14 Data analysis	Page 7
S15 Techniques to enhance trustworthiness	Page 7
**Results/Findings**	
S16 Synthesis and interpretation	Page 8 - 20
S17 Links to empirical data	Page 8 - 20
**Discussion**	
S18 Integration with prior work, implications, transferability, and contribution(s) to the field	Page 20 - 21
S19 Limitations	Page 21 - 22

For peer review only

**Other**	
S20 Conflicts of interest	Page 26
S21 Funding	Page 25

[a]The rationale should briefly discuss the justification for choosing that theory, approach, method, or technique rather than other options available, the assumptions and limitations implicit in those choices, and how those choices influence study conclusions and transferability. As appropriate, the rationale for several items might be discussed together.

For peer review only
